# Impacts of school feeding on educational and health outcomes of school-age children and adolescents in low- and middle-income countries: A systematic review and meta-analysis

**DOI:** 10.7189/jogh.11.04051

**Published:** 2021-09-04

**Authors:** Dongqing Wang, Sachin Shinde, Tara Young, Wafaie W Fawzi

**Affiliations:** 1 Department of Global Health and Population, Harvard T.H. Chan School of Public Health, Harvard University, Boston, Massachusetts, USA; 2 Department of Epidemiology, Harvard T.H. Chan School of Public Health, Harvard University, Boston, Massachusetts, USA; 3 Department of Nutrition, Harvard T.H. Chan School of Public Health, Harvard University, Boston, Massachusetts, USA

## Abstract

**Background:**

School feeding programs are ubiquitous in low- and middle-income countries (LMICs) and may have critical implications for the health and education of school-age children and adolescents. This systematic review aimed to assess the impacts of school feeding on educational and health outcomes of children and adolescents in LMICs.

**Methods:**

Interventional studies on the effects of school feeding on nutritional and health outcomes of children and adolescents receiving primary or secondary education in LMICs were included. MEDLINE, EMBASE, CINAHL, the Cochrane Library, and grey literature were searched (through December 2019) to identify eligible studies. We included randomized controlled trials and controlled before-after studies on school feeding conducted in LMICs among children and adolescents aged 6 to 19 who received primary or secondary education. Two reviewers independently conducted study selection, data extraction, and risk of bias assessment. Meta-analyses were performed for outcomes available in three or more independent studies. Subgroup analyses were conducted by study design and school feeding modality whenever possible.

**Results:**

Fifty-seven articles met the inclusion criteria for the review, including 44 randomized controlled trials and 13 controlled before-after studies; 19 articles were included in the meta-analysis. School feeding resulted in a significant increase in height (mean difference = 0.32 cm; confidence interval (CI) = 0.03, 0.61; *P* = 0.032) and weight (mean difference: 0.58 kg; 95% 95% CI = 0.22, 0.93; *P* = 0.001) over 12 months, compared to those in the control groups. School feeding also resulted in a significant increase in the percentage of school days attended (2.6%; 95% CI = 1.2%, 3.9%; *P* < 0.001).

**Conclusions:**

School feeding is an important approach to improving the health and education outcomes of children and adolescents living in LMICs. More well-designed research is needed to establish further the effectiveness of school feeding for nutritional outcomes and academic achievement.

**Registration:**

PROSPERO ID: CRD42020159003.

Across the developing world, millions of school-age children go to school every day hungry [1]. It is estimated that 73 million children of primary school age living in extreme poverty in 60 countries do not have access to national school feeding programs [1]. Attending classes while hungry severely impacts children’s abilities to learn, thrive, and realize their full educational and health potential as adults [2]. School feeding programs, which provide food to school-going children and adolescents, are ubiquitous worldwide. Most countries have some forms of school meal programs, though in varying formats and scales [3], and it is estimated that 368 million school-aged children and adolescents globally were reached by school feeding programs in 2017 [3]. In low- and middle-income countries (LMICs), it is estimated that some 305 million schoolchildren, approximately half of all those enrolled, eat a daily meal at school; for many of these children, this represents the only regular meal of the day [1]. Therefore, school feeding programs are imperative for the second Sustainable Development Goal (SDG), which entails ending hunger and achieving food security [4].

Besides alleviating hunger, school feeding programs may also increase school attendance, improve academic performance, and generate health, nutrition, and social protection benefits [3,5-7]. Nonetheless, school feeding programs tend to have incomplete coverage in LMICs, where the need for such interventions is greatest [6]. The coronavirus disease 2019 (COVID-19) pandemic has had devastating impacts on people’s livelihoods [8] and has propelled 265 million people worldwide into acute food insecurity [9]. With potentially less food available to children and adolescents at their homes during the pandemic, the role of school meal programs in safeguarding nutrition and education by providing regular and nutritious food will become even more critical. In the meantime, existing determinants of food insecurity remain at regional and household levels, such as household income, household size, household educational level, food production and availability, and food price [10]. School feeding programs play pivotal roles in SDG 3 (ensuring healthy lives and promoting well-being for all at all ages) and SDG 4 (ensuring inclusive and equitable quality education and promoting lifelong learning opportunities for all); it is also highly relevant for SDG 5 (achieving gender equality and empowering all women and girls) since girls dropped out of school disproportionally compared to their male counterparts [4].

Despite being one of the most long-lasting and well-established social protection programs in LMICs, continuous evidence synthesis is needed on the impacts of school feeding on educational and health outcomes in the ever-changing landscape of child and adolescent development. The only Cochrane systematic review on school feeding reported that participants in lower-income countries who were fed at school gained more weight, attended school more frequently, and had better performances in math and short-term cognitive tasks compared to those not fed at school in control groups [11]. However, this Cochrane review was published more than a decade ago and does not include all of the studies currently available [11]. More recent reviews on the benefits of school feeding exist, yet are limited in scope regarding the age range, school level, and the types of outcomes included [12,13]. Therefore, an updated synthesis of the evidence of the impacts of school feeding on a wide array of educational and health outcomes among children and adolescents is imperative.

In this systematic review and meta-analysis, we aimed to assess the impacts of school feeding programs on educational and health outcomes of children and adolescents in LMICs. We focused on educational and health outcomes due to their critical implications to long-term development and well-being and their high relevance to the SDGs. Through this work, our goal was to provide an evidence base that will be informative to the design and implementation of future programs that seek to provide food to school-going children and adolescents in the developing world.

## METHODS

### Eligibility criteria

#### Inclusion criteria

We included studies that met all of the inclusion criteria detailed below. We did not place restrictions on the year, sample size, or duration of the intervention.

1. Randomized controlled trials (RCTs) and controlled before-after studies (CBAs) were eligible. The intervention could be allocated individually or in clusters (classes or schools).
2. Published articles or grey literature were eligible.
3. Studies conducted in low-income, lower-middle-income, or upper-middle-income countries, as defined by the World Bank for the 2020 fiscal year, were eligible.
4. Studies among children and adolescents (girls and boys) aged 6 to 19 years who were receiving primary or secondary education (i.e., primary, middle, or high school) were eligible.
5. Studies that examined the impacts of the provision of food or beverages in the school, including formal meals (breakfast, lunch, or dinner) or snacks, were eligible. We also included food distributed to households conditional on school enrollment and consumed outside the school setting (ie, take-home ration) [6].
6. The comparison (control) group in each study did not receive the school feeding intervention; comparisons among school feeding menus with different food compositions were also eligible.
7. Studies that included at least one outcome of children and adolescents pertinent to education, nutrition, anthropometry, cognition, or morbidity, were eligible. Eligible education outcomes included school enrollment, school attendance, school dropout, grade repetition, and academic performances (math, reading, spelling, vocabulary). Eligible nutrition outcomes included micronutrient status (eg, hemoglobin concentrations and anemia, ferritin concentrations, retinol concentrations) and macronutrient status (eg, blood lipid levels and blood albumin levels). Eligible anthropometry outcomes included height, weight, body mass index, mid-upper arm circumference, skinfold thickness, and bone development (including their z-scores). Eligible cognition outcomes included intelligence quotient, on-task behavior, and memory. Eligible morbidity outcomes included infectious disease diagnoses (eg, malaria and helicobacter pylori infection) or symptoms (eg, fever, cough, diarrhea, and vomiting).

#### Exclusion criteria

We excluded studies with any of the following characteristics.

1. Interventional studies without a proper control group, such as uncontrolled before-after studies, were ineligible.
2. Observational studies were ineligible.
3. Editorials, commentaries, opinions, or review articles were ineligible; these articles, however, were reviewed to identify eligible studies.
4. Studies conducted among preschool children only were ineligible. Feeding interventions among preschool children are important and of great interest but are beyond the scope of this work, which will focus on primary and secondary school settings.
5. Studies that examined the impacts of micronutrient fortification, micronutrient supplementation, or nutrition education were ineligible unless such interventions were complementary to eligible school feeding interventions.
6. Clinical treatment programs targeting patients with medical conditions or exclusively toward individuals with underweight, overweight, or obesity were ineligible.
7. Descriptive studies of school feeding programs without the inclusion of specific outcomes were ineligible.

### Information sources and search strategy

We searched four databases to identify eligible studies, from the inception of each database through December 2019: MEDLINE (via PubMed), EMBASE, CINAHL, and the Cochrane Library. The selection of the electronic databases was made in consultation with a health science librarian with expertise in systematic searching and covered all databases recommended by the *Cochrane Handbook for Systematic Reviews of Interventions* [14]. A search strategy including a combination of keywords, indexing (eg, MeSH terms), and free-text terms, was used. The specific search terms for each database are provided in Table S1 in the **Online Supplementary Document**. We also searched ClinicalTrials.gov and other governmental or organizational websites (World Food Programme, World Health Organization, Food and Agriculture Organization, and World Bank) for studies not identified from the database searching. We conducted a manual search of references included in key articles and previous reviews. We included articles published in English or Chinese. The six articles excluded were in Persian (two articles), Portuguese (one article), or Spanish (three articles). We used EndNote X9 (Clarivate Analytics, Pennsylvania, United States) to store the records retrieved from searches of electronic databases. We imported the records into Covidence (Veritas Health Innovation, Melbourne, Australia) to facilitate streamlined management of the records. Duplicate records were detected and removed by Covidence.

### Study selection

To improve the accuracy of study selection, two reviewers (DW and SS, or DW and TY) independently screened titles and abstracts to assess the initial eligibility of the articles, and irrelevant studies were removed. The full texts of potentially eligible studies were then independently assessed by two reviewers (DW and SS) to confirm eligibility. Disagreements between reviewers were resolved by discussion. Specific reasons for study exclusions were recorded and summarized using the flow diagram for the Preferred Reporting Items for Systematic Reviews and Meta-Analyses (PRISMA) [15].

### Data extraction

To improve the accuracy of the extracted data, two reviewers (DW and SS) independently extracted data from the retained studies and entered data into an extraction form, which was pilot-tested using five randomly selected studies. Discrepancies in data extraction were resolved by discussion. We extracted the following information from each article: title, corresponding authors (name and contact information), journal (or source of information), year of publication, year of intervention, country and setting, source of funding, study design, sample size (number of participants and number of clusters when relevant), age and sex distributions of the sample, intervention (including timing, duration, food and nutritional content, and co-interventions), comparator/control, outcomes assessed, and main findings with point estimates and measures of variance (standard errors, 95% confidence intervals, or *p*-values). We collated multiple reports of a single study as additional results might be available in different articles.

### Risk of bias assessment

Two reviewers (DW and SS) independently evaluated the risk of bias, with any disagreement resolved by discussion. For RCTs, we used version 2 of the Cochrane risk-of-bias tool (RoB 2), which includes five domains for individually randomized RCT [16] and an additional domain for cluster RCT [17]. We judged each domain as “low risk of bias,” “high risk of bias,” or “some concerns.” We considered an RCT to be of low risk of bias if it is judged to have a low risk of bias for all domains; we considered an RCT to be of a high risk of bias if it is judged to have a high risk of bias in at least one domain; we considered an RCT to have some concerns if it raises some concerns in at least one domain but is not of a high risk of bias for any domain [16].

For CBAs, we used the Risk of Bias in Non-randomized Studies of Interventions (ROBINS-I) tool, which considers biases from seven domains [18]. We judged each domain as “low risk of bias,” “moderate risk of bias,” “serious risk of bias,” “critical risk of bias,” or “no information.” We considered a CBA to be of low risk of bias if it is judged to have a low or moderate risk of bias for all domains; we considered a CBA to be of a high risk of bias if it is judged to have a serious or critical risk of bias in one or more domains; we considered a CBA to have some concerns if the assessment has no information for one or more domains but is of low or moderate risk of bias for all other domains.

### Data synthesis

We summarized the characteristics and key findings of all included studies in the form of a summary table. Random-effects, inverse-variance-weighted meta-analyses were conducted for outcomes included in three or more independent studies with a sufficiently consistent definition of the outcome and the timing of the outcome assessment. As a result, the meta-analyses included nine outcomes, including 1) height; 2) height-for-age Z-score (HAZ); 3) weight; 4) weight-for-age Z-score (WAZ); 5) body mass index-for-age Z-score (BAZ); 6) hemoglobin concentrations; 7) plasma/serum ferritin concentrations; 8) mathematical or arithmetic skills; and 9) school attendance. We used the random-effects method because the effect of school feeding is presumed to be heterogeneous across time and populations, and also because the menus of previous school feeding interventions have been quite heterogeneous.

We treated school feeding as a dichotomous exposure. Effect estimates for continuous outcomes were expressed as mean differences with 95% confidence intervals (CIs) comparing the intervention group with the control group. To account for the different durations of the intervention across studies, we converted the estimates to the differences between the two groups over a period of 12 months using the monthly difference between arms. School attendance was standardized across studies as the percentage of possible school days attended in each study. When two or more relevant school-feeding arms (eg, in-school feeding and take-home ration, or different school feeding menus) were present, the average effects across arms were used. When cluster-based studies did not account for the clustered design in the analysis, we used the extracted average cluster size and an intracluster correlation coefficient (ICC) to correct the variance estimate using the approach recommended in the Cochrane Handbook for Systematic Reviews of Interventions [14]. Based on values recommended or commonly used in previous literature [11,19,20], the ICC values used to correct the variance estimate were 0.016 for HAZ, 0.025 for weight, WAZ, BAZ, hemoglobin concentrations, and plasma/serum ferritin concentrations, and 0.15 for attendance and mathematical/arithmetic skills.

We assessed the robustness of the results by removing each study from the meta-analyses (ie, the “one-study-removed” procedure). Whenever possible (ie, when at least two studies were available in each subgroup), we conducted subgroup analyses by study design (RCT or CBA) and modality of intervention (formal meals or snacks) to assess the sources of heterogeneity of the effects. We did not conduct subgroup analyses by risk of bias as most of the included studies had a high risk of bias. Other subgroup analyses we considered that were not ultimately feasible due to the small number of studies or lack of subgroup-specific results included the presence of co-interventions (by itself or combined with complementary interventions), year of study, country or region, level of food insecurity of the region, age group of participants (primary or secondary education), sex of participants, and type of report (published or unpublished). We used funnel plots to detect publication bias for outcomes available in at least five studies. We conducted meta-analyses using Comprehensive Meta-Analysis Software Version 3 [21] using a two-sided level of 0.05.

### Assessment of certainty of evidence

The overall certainty of evidence for each outcome included in the meta-analyses was assessed using the Grading of Recommendation, Assessment, Development, and Evaluation (GRADE) approach that considers risk of bias, publication bias, imprecision, inconsistency, and indirectness [22-27]. The evidence for each outcome was accordingly judged as high, moderate, low, or very low [22].

### Registration and reporting

The full protocol of this work was published elsewhere [28]. This work was registered with the International Prospective Register of Systematic Reviews (PROSPERO ID: CRD42020159003). We conducted and reported this study in accordance with the Preferred Reporting Items for Systematic Review and Meta-Analysis Protocols (PRISMA) [15].

## RESULTS

### Basic characteristics of the included studies

**Figure 1:**
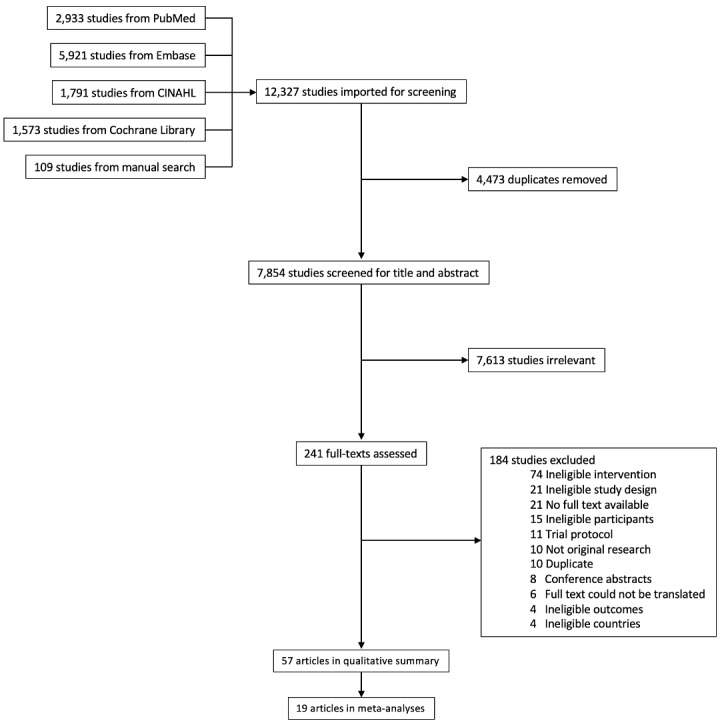
PRISMA flow diagram.

We identified 7854 articles from four electronic databases and a manual search of references of key articles, previous reviews, and grey literature. Among the retrieved articles, 7613 were judged as irrelevant and excluded after the title and abstract screening. Among the 241 articles included in the full-text review, 184 were excluded due to various exclusion criteria, resulting in 57 articles included in the data synthesis (**Figure 1**).

Among the 57 retained articles, 44 and 13 were based on RCTs [29-72] and CBAs [73-85], respectively. The 57 articles arose from 39 independent studies, of which 26 were RCTs and 13 were CBAs, and most (12 RCTs and 10 CBAs) allocated the intervention by school. The majority of the studies included a separate control group that did not receive school feeding, with four studies [30,33,38,48] employing a crossover design where the same individuals received both the intervention and the control. Five studies each were conducted in China [38,43,61,62,82] and India [48,54,58,72,76], three in Jamaica [30,36,74], two each in Ghana [69,71], Indonesia [35,73], Iran [57,81], Kenya [39,80], Peru [32,33], Philippines [37,65], South Africa [68,77], Uganda [50,83], and Vietnam [53,78], and one each in Bangladesh [84], Benin [79], Burkina Faso [52], Colombia [75], Lebanon [85], Papua New Guinea [29], Senegal [67], and Thailand [55].

Seventeen studies focused on the provision of in-school snacks [39,43,53,54,57,58,62,65,69,73,78,80-85], eight on the provision or modification of in-school breakfast [30,32,33,36,38,61,74,77], seven on the provision or modification of in-school lunch [55,67,68,71,72,75,76], and seven included more than one modality of school feeding [29,35,37,48,50,52,79]. In-school meals were prepared either centrally in one or a handful of kitchens or a decentralized manner in each school. The distribution of take-home rations was involved in two studies [50,52]. For in-school meals, the compliance was often monitored through direct supervision by teachers or study staff when students consumed the food. Details on the costs of food or the costs of operation were rarely provided.

Most of the studies used school feeding as the sole intervention without any complementary interventions (to all participants or those in the intervention arms), with the exception of three studies that provided deworming [61,63,68], four that provided nutrition or health education [72,80,81,85], and one that provided both deworming and nutrition education [78]. The duration of intervention ranged from one week to 2 years. Two studies were conducted among secondary school students [74,79], three studies among both primary and secondary school students [54,72,81], and the rest 34 studies were among primary school students. The sample size for each study ranged from 39 to 4236 students. Four studies were conducted among girls only [43,54,75,79], one study among boys only [73], and the remaining 34 studies among girls and boys. Ten studies were conducted among children of middle childhood (6 to 9 years) [30,38,48,53,57,69,76,78,80,83], 10 studies among adolescents (10 to 19 years) [43,54,55,62,67,68,72,74,79,85], and the remaining 18 studies including both children and adolescents; the age distribution for one study was not reported [37]. Most of the studies were conducted in governmental/public schools. The main characteristics of the included studies are provided in **Table 1****.**

**Table 1:** Characteristics of the included studies

Reference	Study design (unit of allocation)	Country	Participants in analysis	Age at baseline	Modality	Intervention	Frequency	Duration	Comparison	Outcomes	Key findings
Bailey 1962 [73]	CBA (individual)	Indonesia	504 boys in primary school	7 to 13 y	In-school snacks	Palm-sugar, Green gram and palm sugar, Skimmed milk (powder), Saridele (powder), Tempeh (soya), Tempeh (velvet bean)	Not mentioned	12 mo	Iron supplementation (100 mg/d)	Weight increment; Height increment; Hb (% increment)	Weight gains did not differ between groups; height gains were slight and not greater than expected.
Lampl 1978 [29]	RCT (individual)	Papua New Guinea	86 primary school students	7.7 to 13.0 y	Food added to habitual diet	(1) 10 g additional protein as skim milk or milk powder, offered as a milk drink or mixed with the evening meal. (2) 20 g additional protein as skim milk or milk powder, offered as a milk drink or mixed with the evening meal.	Daily on school days, (5 d/week)	8 mo	Habitual diet in school	Height; Weight; Periosteal breadth; Endosteal breadth; Compact bone breadth; Bone maturity score; Skeletal age; TSF; Subscapular skinfold; Z-score of height increment; Z-score of bone maturity score increment	Supplemented children showed increased growth in height, weight, and periosteal bone breadth and increased increments of skeletal maturation.
Powell 1983 [74]	CBA (unit of allocation unclear)	Jamaica	108 adolescent students	11 to 17 y	In-school breakfast	Half-pint of milk and either a banana cake or a patty (pastry containing minced meat and vegetables).	Daily on all school days	Two school terms	No intervention	Weight (% expected for age); Height (% expected for age); Arithmetic; Spelling; Reading; Attendance	No improvement was found in weight-for-age; small benefits were found in attendance and school achievement.
Spurr 1987 [75]	CBA (school)	Colombia	39 malnourished primary school girls	8 to 11 y	In-school lunch	The opening of a school cafeteria which provided a daily hot lunch; the menu provided a weekly supplementation of 2900 to 3000 kcal, which amounted to an average of 600 kcal/d, consisting of 68% carbohydrate, 12% protein, and 20% fat.	Daily on school days (5 d/week)	4 to 5 mo	No intervention	Height; Weight; TSF; Sum skinfolds (triceps + subscapular + abdominal); MUAC; Upper arm muscle area; Head Circumference; Basal metabolic rate; Maintenance energy expenditure; Total daily energy expenditure; Energy expenditure in activity; Undernutrition	Skinfold thickness, mid-arm circumference, and weight gain velocity were higher after dietary supplementation.
Agarwal 1989 [76]	CBA (school)	India	450 primary school students	6 to 8 y	In-school lunch	Three well-accepted recipes providing around 450 to 500 kcal with 10 to 12 g protein.	Daily on school days	2 y	No intervention	Annual height gain; Annual weight gain; Full-scale IQ; Verbal IQ; Performance IQ; Arithmetic test score	Children receiving supplementation showed slight increments in full scale, verbal, and performance IQ; no effect was observed on physical growth.
Chandler 1995 [30]; Chang 1996 [31]; Grantham-McGregor 1998 [34]	Crossover RCT (class)	Jamaica	197 primary school students	9 to 10 y (mean: 9.3 y)	In-school breakfast	Breakfast consisted of 225 mL of chocolate milk and a cheese sandwich (68 g bread and 28 g cheese), providing 2147 kJ and 21.3 g protein.	Daily on all school days	The feeding commenced in the schools 1 week before testing began and continued until the last test was completed	A quarter of an orange (30 g) providing 63 kJ energy and 0.3 g protein as a ‘placebo’	Attention (visual search); Memory (backward digit span); Verbal fluency; Information processing [30]	Verbal fluency of undernourished children improved when they received breakfast, whereas that verbal fluency in the adequately nourished children did not change. There were no other effects of breakfast on test scores. School breakfast may only benefit children's behavior in the presence of adequate classroom infrastructure.
										Recorded classroom behaviors (attention on task, talking to another child; gross motor movements; participation in the class) [31]	
										Verbal fluency [34]	
Jacoby 1996 [32]	RCT (school)	Peru	352 primary school students	Treatment group: Mean: 11.4 y; SD: 1.5 y; Control group: Mean: 11.6 y; SD: 1.7 y	In-school breakfast	Four cookies and an instant drink; periodically alternated with a cake and drinks of different flavors and similar nutritional content.	Daily	About 20 d	No intervention	Attendance; Visual perceptual organization and visual-motor coordination (coding test); Reading; Vocabulary; Math	The intervention improved school attendance.
Pollitt 1996 [33]	Crossover RCT (individual)	Peru	54 primary school students	9 to 11 y	In-school breakfast	A small cake (80 g) and a glass of a beverage similar in taste and color to milk (50 g).	Not mentioned	Not mentioned	A diet soda without caffeine as a placebo when the subjects were scheduled not to receive breakfast	Number discrimination; Peabody picture vocabulary test; Raven's Progressive Matrices; Reaction time test; Sternberg memory search test; Stimulus discrimination test	Short-term memory scanning was slower with the placebo than with the breakfast for at-risk children but not for no-risk children. No-risk children showed more rapid visual stimulus discrimination under the placebo than under the breakfast, whereas no such effect was observed in the at-risk group.
Richter 1997 [77]	CBA (school)	South Africa	108 primary school students	Intervention group: Mean: 10.5 y; SD: 1.9 y; Range: 7 to 14 y; Control group: Mean: 8.3 y; SD: 0.8 y; Range: 7 to 10 y	In-school breakfast	30 g corn flakes served with 100 ml skim milk and a banana.	Daily on school days	6 weeks without interruption by school holidays	No intervention	WISC-A; WISC-B; WISC digit; Attention (vigilance); Teacher-rated behaviors (attention, hyperactivity, social skills, oppositional); Occurrence and duration of videotaped behavior	The intervention had a beneficial effect on the cognitive and behavioral performances of socially disadvantaged, undernourished children in their first 2 y of school.
de Pee 1998 [35]	RCT (individual)	Indonesia	188 primary school students	7 to 11 y	In-school breakfast and lunch	(1) Vegetable group: carotene-rich, dark-green, leafy vegetables and carrots. (2) Fruit group: carotene-rich fruit. (3) Retinol-rich group: retinol-rich protein sources	2 meals/d, 6 d/week	9 weeks	Low-retinol, low-carotene group: food low in both retinol and carotene	Beta-Carotene; Beta-Cryptoxanthin; Alpha-Carotene; Lutein; Lycopene; Zeaxanthin; Hb; Hematocrit; Serum ferritin; Serum transferrin receptor	This study challenges the assumption that 6μg dietary beta-carotene provides one retinol equivalent, which has implications for choosing strategies for controlling vitamin A deficiency.
Powell 1998 [36]	RCT (individual)	Jamaica	791 primary school students	107.6 ± 14.7 mo	In-school breakfast	A cheese sandwich or spiced bun and cheese and flavored milk	Daily on all school days	One school year	One-quarter of orange before the start of classes	Arithmetic; Spelling; Reading; Attendance; Height; Weight; BMI	School breakfast produced small benefits in children's nutritional status, school attendance, and achievement.
Tan 1999 [37]	RCT (school)	Philippines	1974 primary school students	Not mentioned	In-school meals	All pupils in beneficiary schools received a free school meal while classes were in session	Daily on school days	Two school years	No intervention	Dropout; Math score; Filipino score; English score	In the schools with a feeding program, dropout rates decline 2.9% compared with a decline of 1.2% in control schools.
Ma 1999 [38]	Crossover RCT (individual)	China	151 primary school students	Approximately 8 y	In-school breakfast	High-energy breakfast: Cornflakes (or sugar-coated cornflakes), milk, bread, and peanut butter, providing energy and protein that met or exceeded 25% of RDA	Daily for 5 successive days	5 successive days	Low-energy breakfast: steamed bread, rice porridge, bread, and fermented cucumbers, providing energy and protein less than 10% of RDA	Cognitive performance (addition, multiplication, number checking, logic, creativity); Physical endurance; Self-perception	No effects of breakfast on any performance indicator.
Siekmann 2003a [39]; Siekmann 2003b [40]; Whaley 2003 [41]; Grillenberger 2003 [42]; Sigman 2005 [44] Neumann 2007 [47]; Omwami 2011 [56]; Neumann 2013a [63]; Neumann 2013b [64]; Hulett 2014 [66]	RCT (school)	Kenya	555 primary school students [39]	6 to 14 y (median: 7.4 y) [39]	In-school snacks	Githeri + meat (Meat group)	Daily on all school days (5 d per week)	10 mo [39]	No intervention	Hb; Plasma ferritin; Serum iron; Serum zinc; Serum copper; Plasma vitamin B-12; Plasma folate; Plasma retinol; RBC riboflavin [39]	Supplementation with small amounts of meat or milk reduced the prevalence of vitamin B-12 deficiency [39].
			199 primary school students [40]	6 to 14 y (median: 7.4 y) [40]				One academic year [40]		*Helicobacter pylori* IgA; *Helicobacter pylori* IgG; *Helicobacter pylori* IgM; Transformed rotavirus; Tetanus toxoid IgG; Recombinant malaria antibody [40]	Compared with the control subjects, only the group eating meat had an increase in H. pylori IgM antibodies [40].
			542 primary school students [41]	Mean: 7.63 y [41]		Githeri + milk (Milk group)		21 mo (7 school terms) [41]		Raven’s Progressive Matrices; Verbal meaning; Arithmetic tests [41]	Supplementation with animal source food has positive effects on cognitive performance. These effects are different across domains of cognitive functioning and different forms of animal source foods [41].
			498 primary school students [42]	6 to 9 y; Mean: 7.1 y; SD 0.8 y [42]				18 mo [42]		Weight; Height; WHZ; HAZ; MUAC; TSF; Subscapular skinfold; mid-upper-arm muscle area; Mid-upper-arm fat area [42]	Food supplements had a positive impact on weight gain. The addition of meat increased lean body mass [42].
			Approximately 540 primary school students [44]	7 to 8 y [44]				21 mo (7 school terms) [44]		Playground activities (high activity, low activity; leadership, initiates, solitary play) [44]	Supplemented children were more active and showed more leadership behavior and initiative than non-supplemented children [44].
			900 primary school students [47]	6 to 14 y (median: 7.4 y) [47]				21 mo (7 school terms) [47]		Raven’s Progressive Matrices; Behaviors during free play; End-of-term test scores [47]	The meat group showed the steepest rate of increase on Raven’s Progressive Matrices scores and in total and arithmetic test scores. The plain githeri and meat groups performed better over time than the Milk and Control groups on arithmetic tests. The Meat group showed the greatest increase in percentage time in physical activity and leadership behaviors [47].
			444 primary school students [56]	6 to 14 y (median: 7.4 y) [56]				21 mo (7 school terms) [56]		Attendance [56]	The intervention groups performed better than the control group on the repeated measure of school attendance [56].
			902 primary school students [63]	Mean: 7.3 y; SD 1.1 y [63]				26 mo [63]		Total illness; Severe illness; Malaria; Fever; Chills; Poor appetite; Reduced activity; Upper respiratory infection; Ear infection; Gastroenteritis; Typhoid; Jaundice; Sore mouth; Skin infection; Eye problems [63]	Beneficial effects of both animal-source foods and of vitamin A-fortified oil on morbidity outcomes [63].
			910 primary school students [64]	6 to 14 y (median: 7.4 y) [64]				26 mo [64]		MUAC (cm/mo); MAMA (mm^2^/mo); Height (cm/mo); Weight (kg/mo); TSF (mm/mo); MAFA (mm^2^/mo) [64]	The meat group showed the steepest rates of gain in MUAC and MAMA over time. The meat group showed the least increase in TSF and MAFA of all groups [64].
			360 primary school students [66]	6 to 9 y; Mean: 7.1 y; SD 0.8 y [66]		Basic githeri with additional vitamin A-fortified oil equivalent to the energy provided in the Meat and Milk groups (Energy group)		19 mo (5 school terms) [66]		Arithmetic test score; Total test score [66]	Greater improvements in test scores of children receiving animal source foods [66].
Du 2004 [43]; Zhu 2005 [45]; Zhu 2006 [46]; Zhu 2008 [49]	RCT (school)	China	681 adolescent girls in primary school [43]	10 y	In-school morning snacks	Milk + Ca group: 330 ml ultra-heat-treated milk, which had been fortified to contain 560 mg calcium.	Daily (5 d/week) except weekends and holidays	24 mo	Habitual diets without supplementary milk	Height; Sitting height; Weight; BMC; size-adjusted BMC; Bone area; Bone mineral density [43]	The milk fortified with cholecalciferol improved vitamin D status compared with the milk alone or control groups [43].
			606 adolescent girls with bone radiograph data, and 128 adolescent girls with biochemical data [45]			Milk + Ca + VitD group: 330 ml ultra-heat-treated milk fortified with 560 mg calcium and 5 or 8 mg of cholecalciferol.				Periosteal diameter; Medullary diameter; Combined cortical thickness; Length of the second metacarpal; BAP; IGF-1; PTH [45]	Milk supplementation showed positive effects on periosteal and endosteal apposition of cortical bone [45].
			501 adolescent girls in primary school [46]							Percentage changes (3 y after intervention withdrawal) in height, sitting height, weight, BMI, total-body BMC, total-body bone area, total-body BMD, and size-adjusted total-body BMC [46]	Milk supplementation during early puberty does not have long-lasting effects on bone mineral accretion [46].
			345 adolescent girls in primary school [49]							Total body size-corrected BMD; Arms size-corrected BMD; Legs size-corrected BMD; Midriff size-corrected bone BMD; Pelvis size-corrected BMD [49]	Milk supplementation showed positive effects on bone mineral accretion when accounting for the changing skeletal size during growth, and the effects were mainly on the lower limbs [49].
Hall 2007 [78]	CBA (school)	Vietnam	1080 primary school students	6 to 7 y	In-school snacks	A 30-g packet of baked biscuits made from wheat flour fortified with 18 vitamins and minerals that provided 150 kcal of energy. The students were also given 200ml of ultra-heat-treated cow’s milk fortified with vitamins A and D, which also provided 150 kcal of energy.	Daily on school days (5 d a week)	17 mo	No intervention (In order to provide some benefits to comparison schools, all children were dewormed with albendazole)	Weight; Height; HAZ; WAZ; WHZ; BMI	The intervention had a small effect on weight gain, but undernourished children benefited the least.
Muthayya 2007 [48]	Crossover RCT (individual)	India	69 primary school students	7 to 9 y	In-school breakfast, lunch, and mid-morning snacks	(1) Small breakfast (187 kcal), snack (153 kcal), and standard lunch (500 kcal). (2) Standard breakfast consisted of a traditional Indian meal of wheat-flour bread with clarified butter and potato curry. The snack was a mango-flavored co-extruded bar. Lunch consisted of a traditional lunch of spiced vegetable rice, chickpea curry, and a vermicelli dessert. (3) Standard breakfast (340 kcal) plus a snack (153 kcal) plus a small lunch (347 kcal)	During one specific intervention day for each intervention (each intervention lasted for one week)	The children received three different interventions, each one week apart	Standard breakfast (340 kcal) plus standard lunch (500 kcal)	Memory (immediate picture recognition and delayed picture recognition); Continuous performance task; Psychomotor speed (finger tapping)	A more evenly distributed energy intake throughout the morning by consuming a mid-morning snack improves memory performance even when the total amount of energy consumed during the morning is not altered.
Alderman 2008 [50]; Adelman 2008 [51]; Alderman 2012 [59]; Adelman 2019 [70]	RCT (school)	Uganda	Primary school students (sample size unclear and likely varied by outcome)	6 to 14 y	In-school lunch and mid-morning snacks	The lunch consists mainly of beans and either hot posho (maize meal) or rice. The lunch also includes vegetable oil and salt. The snack consists of a porridge made from micronutrient fortified corn-soy-blend, sugar, and water.	Daily on all school days	18 mo	No intervention	Enrollment; School attendance in the morning; School attendance in the afternoon; Age at entry to primary school; Number of grades repeated [50]	Both in-school feeding and THR resulted in higher school attendance, with impacts varying by grade and gender. Both programs reduce grade repetition, but in-school feeding’s impacts are larger. In-school feeding also reduced girls’ age at entry [50].
					THR	THR qual in size and composition to the in-school meals. The rations are provided to THR beneficiary households once per month. THR beneficiary households receive a THR for each primary-school-age child enrolled and attended school at least 80% of the days in the previous month.	Monthly			Math score; Literacy score; Raven’s Colored Progressive Matrices; Attention (forward digit span); Memory (backward digit span) [51]	Neither program had an impact on the math and literacy test scores of the 6-to-14-y-old. However, the THR program increased math scores of the 11-to-14-y-old, and in-school feeding slightly increased test scores of the 11-to-14-y-old. Both programs improve cognitive function [51].
										Enrollment; Morning school attendance; After school attendance; Grade repetition [59]	Positive impacts of the in-school feeding program on primary school enrollment [59].
										Any anemia; Moderate-to-Severe Anemia [70]	The programs reduced any anemia and moderate-to-severe anemia in primary-school-age adolescent girls [70].
Kazianga 2009 [52]; Kazianga 2012 [60]	RCT (school)	Burkina Faso	4236 elementary school students (specific numbers varied by outcomes)	6 to 15 y	In-school lunch	Not described	Daily on all school days	One academic year	No intervention	Attention (forward digit span); Memory (backward digit span); BAZ; WAZ; Hb [52]. Enrollment; Learning outcomes: Answers to math questions; Cognitive abilities: Raven's Progressive Matrices; Attendance; Participation in child labor [60]	Both interventions increased enrollment. The scores on mathematics improved for girls in both programs. The interventions also led to an adjustment in child labor, with children shifting away from on-farm labor and off-farm productive task [52,60].
					THR	10 kg of cereal ﬂour per month, conditional on a 90% attendance rate	Monthly				
Alaofe 2009 [79]	CBA (school)	Benin	68 adolescent girls in secondary boarding schools	12 to 17 y	In-school breakfast, and improved menu to in-school lunch and dinner	Iron-containing foods (beef, liver, poultry, and lentils) and foods known to enhance nonheme iron absorption (fish and fruits) were increased, whereas foods known to inhibit nonheme iron absorption (eg, coffee) were decreased. Food-preparation techniques such as soaking of dried beans to reduce the phytate content and avoid vitamin C destruction by cooking foods for shorter periods and in less water were established. The ultimate goal of the study was to increase the content of absorbable iron to 1.90 mg/d at the lowest cost possible.	Daily	22 weeks	Habitual meals	Low serum ferritin; Low serum iron; High TIBC; Low transferrin saturation; Low Hb	A multi-dietary strategy aimed to improve available dietary iron can reduce iron-deficiency anemia in adolescent girls.
Lien 2009 [53]	RCT (school)	Vietnam	444 primary school students	7 to 8 y	In-school snacks	Fortified milk: Two servings of 250-ml milk fortified with micronutrients. Regular milk: Two servings of 250-ml regular milk.	Each school day (6 d a week) for six months, except for holidays	6 mo	No intervention	Weight; Height; WAZ; Underweight; HAZ; Stunting; WHZ; Wasting; Hb; Anemia; Ferritin; Vitamin A; Zinc; Zinc deficiency; Urine iodine; Urine iodine deficiency	Milk consumption benefited the children in rural Vietnam, including lowering the occurrence of underweight and stunting, improving micronutrient status, and better learning indicators.
Ohiokpehai 2009 [80]	CBA (school)	Kenya	102 primary school students	6 to 9 y	In-school snacks	Mid-morning corn-soy blend porridge: 100 g corn-soy blend as porridge per day for five months with a THR of 500 g for the two-day weekend.	Daily (including weekend)	5 mo	Mid-morning corn-pigeon pea blend porridge: 100 g corn-pigeon pea blend as porridge with a 500-g THR for the weekend	Stunting; Underweight; Wasting; WAZ; HAZ; WHZ	Improvements in school children's nutritional indicators under both intervention and control programs indicate that both crops (soybean and pigeon pea) can be used to improve nutritional status.
Tupe 2009 [54]	RCT (individual)	India	111 adolescent girls in primary and secondary school	10 to 16 y	In-school snacks	Freshly prepared snacks designed using the girls' habitual food items along with zinc-rich foods and by modifying cooking methods to enhance zinc bioavailability.	Daily on school days (6 d/week)	10 weeks	No intervention	Hb; Plasma zinc; Simple reaction time; Recognition reaction time; Memory; Raven’s Standard Progressive Matrices; Taste acuity	Supplementation of ayurvedic zinc and zinc-rich foods improved cognitive performance and the recognition threshold for salt of adolescent girls.
Mayurasakorn 2010 [55]	RCT (individual)	Thailand	387 adolescents in primary school	10 to 12 y	Food added to in-school lunch	10-egg group: 2 boiled eggs on every Monday through Friday	Daily on school days (5 d a week)	12 weeks	3-egg group: One boiled egg every Monday, Wednesday, and Friday	Total cholesterol; Triacylglycerols; HDL-C; LDL-C; TC:HDL; Albumin; Prealbumin	No difference in any biochemical indicators between 3 eggs/week group and 10 eggs/week group. Total Cholesterol, LDL, and TC:HDL decreased. Albumin, prealbumin, and HDL levels increased.
Li 2012 [61]	RCT (individual)	China	228 primary school students	6 to 11 y	Food added to in-school breakfast	(1) Three meals at school with 2 g spirulina in breakfast as cakes, sweet rice dumplings, or rice noodles. (2) Three meals at school with 4 g spirulina in breakfast as cakes, sweet rice dumplings, or rice noodles.	Daily on all school days	10 weeks	Habitual diet (three meals at school with no spirulina)	Serum retinol; Serum β-carotene; Total body stores of vitamin A	Spirulina may increase the total-body vitamin A stores of Chinese school-age children.
Rahmani 2011 [57]	RCT (school)	Iran	469 primary school students	Boys: Mean 7.9 y; SD: 0.89 y. Girls: Mean 7.5 y; SD: 0.95 y	In-school snacks	Tetra-pack (250 ml) sterilized and homogenized milk distributed daily at mid-morning, with a fat content of 2.5%.	Daily	3 mo	No intervention	Weight; Height; Mid-arm circumference; IQ based on Raven’s Progressive Matrices; Verbal score; Non-verbal test score based on WISC; IQ score; GPA	School feeding programs focusing on milk supplementation had beneficial effects on physical function and school performances among girls in Iran.
Vaz 2011 [58]	RCT (individual)	India	287 primary school students	7 to 10.5 y	In-school snacks	Fortified choco-malt beverage powder: 40 g daily fortified choco-malt beverage providing ~ 158 kcal (662 kJ), 3.2 g protein, 1.4 g fat, and 19 key micronutrients.	Daily	120 d (4 mo)	No intervention	Physical performance measures; RBC riboflavin; Plasma pyridoxal; Plasma vitamin B-12; RBC folate; RBC thiamin; RBC niacin; Serum vitamin C; Plasma ferritin; Serum CRP; Serum transferrin receptor	There were within-subject increases in aerobic capacity and whole-body endurance accompanied by improvements in the status of iron thiamin, riboflavin, pyridoxal phosphate, folate, and vitamins C and B-12 in the fortified group compared to the within-subject changes in the other two groups.
						Energy-equivalent unfortified placebo: 40 g daily unfortified choco-malt beverage providing ~ 158 kcal (662 kJ), 3.2 g protein, and 1.4 g fat.					
Kleiman-Weiner 2013 [62]	RCT (school)	China	2280 adolescents in elementary school	10 to 11 y	In-school snacks	(1) Chewable vitamin. (2) Eggs.	Daily on school days	6 mo	No intervention	Hb; Math test score	There was no effect of eggs on Hb levels or math test scores.
Joulaei 2013 [81]	CBA (individual)	Iran	2897 primary and secondary school students	7 to 13 y	In-school snacks	A snack package with traditional, diverse, and nutritious foods, combined with education and media campaigns on nutrition and lifestyle.	Unclear	2 y	Education and media campaign without free snacks	BMI for age (severe thinness; thinness; overweight; obesity); BMI (mild malnutrition; moderate and severe malnutrition); Weight; Height	The prevalence of low BMI decreased after intervention among girls but not among boys.
Cervo 2014 [65]	RCT (individual)	Philippines	98 elementary school students	6 to 12 y; Mean: 8.44 y; SD: 0.20 y	In-school snacks	(1) One can (140 g) of pineapple providing 76 kcal of energy. (2) Two cans (280 g) of pineapple providing 152 kcal of energy	Daily (including weekends)	9 weeks	No canned pineapples	Hb; innate immunological markers; adaptive immunological markers;	Intake of both 1 can and 2 cans of pineapple may shorten the duration and incidence of infection and increase the production of granulocytes and immunological cells.
Diagne 2014 [67]	RCT (school)	Senegal	Approximately 2900 adolescents in primary school	10 to 11 y	In-school lunch	120 g cereals (maize), 30 g legumes, 20 g enriched oil, and 5 g iodized salt	Daily on all school days	13 mo	No intervention	Cognitive acquisitions; Grade repetition; Dropout	School canteens improved cognitive abilities and reduced dropout and grade repeat rates.
Lin 2015 [82]	CBA (school)	China	892 primary school students	6 to 13 y	In-school snacks	Salty egg (50 g) and ultra-high-temperature-sterilization school milk (200 g).	Daily on school days	1 y	No intervention (habitual school diet)	Height; Weight; Undernutrition; Lean body mass; Body fat	Among boys, the increase in weight, increase in lean body mass, and decrease in malnutrition rate were all greater in the intervention group compared with the control group.
van der Hoeven 2016 [68]	RCT (individual)	South Africa	167 primary school students	6 to 12 y	In-school lunch	300 g cooked African leafy vegetable dish and school meal starch.	Daily on school days (5 d/week)	62 school days	Normal school meal of the same starch portion accompanied by a serving spoon of relish including vegetables or legumes and sometimes meat or soya mince	Hb; Serum ferritin; Serum transferrin receptor; Zinc protoporphyrin; CRP; Serum zinc; Serum retinol	African leafy vegetables were unable to improve serum retinol, serum ferritin, or Hb if there were only mild deficiencies. African leafy vegetables did not improve serum zinc despite the low zinc status in the study population.
Baum 2017 [83]	CBA (school)	Uganda	241 primary school students	6 to 9 y	In-school snacks	(1) Two eggs per day for five days per week. (2) One egg per day for five days per week.	Daily on all school days	6 mo	No eggs	Height; weight; TSF; MUAC	Egg supplementation can improve parameters of growth in school-aged children participating in school feeding programs.
Adams 2017 [84]	CBA (school)	Bangladesh	351 primary school students	6 to 11 y	In-school snacks	A packet of fortified biscuit (75 g) a range of micronutrients contributing to about 75% of the daily requirements of vitamin A, folate, iron, iodine, zinc, and magnesium.	Daily on all school days	One year	No intervention	Hb; ferritin; folic acid; B12; retinol; zinc; urinary iodine; vitamin D; anemia; iodine deficiency; vitamin D deficiency; zinc deficiency	Daily consumption of fortified biscuits by primary school children had a positive impact on levels of iron, folic acid, vitamin B12, retinol, and vitamin D. Levels of anemia and vitamin D deficiency were also reduced.
Lee 2018 [69]	RCT (individual)	Ghana	939 primary school students	6 to 9 y	In-school snacks	(1) Skimmed-milk powder providing 8.8 g milk protein with the addition of 0.2 g multiple micronutrient powder. (2) Skimmed-milk powder providing 4.4 g milk protein with the addition of 0.2 g multiple micronutrient powder. (3) Skimmed-milk powder providing 4.4 g milk protein and 4.4 g rice protein with the addition of 0.2 g multiple micronutrient powder.	Daily on school days	An entire academic year (about 9 mo)	0.2 g multiple micronutrient powder blended in a small amount of sucrose	WAZ; HAZ; MUAC; Fat-free mass index; Fat mass index; Pattern recognition memory; Attention	The consumption of 8.8 g milk protein/d improved executive cognitive function compared with other supplements and led to the accretion of more lean body mass, but not more linear growth.
El Harake 2018 [85]	CBA (school)	Lebanon	183 adolescents in primary school	10 to 12 y	In-school snacks	Locally prepared nutritious snacks: Children in the intervention group were provided with one snack item daily during the school break according to a pre-planned weekly menu. Snacks consisted of cheese sandwiches, spinach pies, or thyme pastries. Children were also offered fruits (oranges, apples, or bananas) twice a week, depending on seasonality, availability, and cost. There were also classroom-based health and nutrition education modules on observational learning, behavioral capability, and self-efficacy.	Daily	6 mo	Usual curriculum and a standard snack	BAZ; HAZ; WAZ; Waist to height ratio	A positive impact of this school-based nutrition intervention on dietary knowledge, attitude, and nutritional status of Syrian refugee children.
Gelli 2019 [71]	RCT (households and schools)	Ghana	3133 primary school students (specific numbers varied by outcomes)	5 to 15 y	In-school lunch	School lunch menus that met ∼ 30% of the recommended daily intake for children aged 6 to 12 y and included foods grown by farmers in the community and the broader agroecological zone.	Daily on school days	1 y	No intervention	HAZ; BAZ	No effect of the school meals on HAZ and BAZ in children aged 5 to 15 y.
Anitha 2019 [72]	RCT (school)	India	243 adolescents in primary and secondary school	10 to 14 y	In-school lunch	Millet-based lunch	Daily on all school days	3 mo	Fortified rice-based lunch	HAZ; BAZ	Improvement in stunting and the body mass index in the intervention group but not in the control group.

BAP – bone alkaline phosphatase, BAZ – body mass index for age z-scores, BMC – bone mineral content, BMD – bone mineral density, BMI – body mass index, CBA – controlled before-after study, CRP – C-reactive protein, GPA – grade point average, HAZ – height-for-age Z-score, Hb – hemoglobin, HDL-C – high-density lipoprotein cholesterol, IGF-1 – insulin-like growth factor 1, IQ - intelligence quotient, LDL-C – low-density lipoprotein cholesterol, MAFA – mid-upper-arm fat area, MAMA – mid-upper-arm muscle area, MUAC – mid-upper arm circumference, PTH – parathayroid hormone, RBC – red blood cell, RCT – randomized controlled trial, RDA – recommended dietary allowance, SD – standard deviation, TC:HD – ratio of total cholesterol to HDL cholesterol, THR – take-home ration, TIBC – total iron binding capacity, TSF – triceps skinfold, WAZ – weight-for-age Z-score, WHZ – weight-for-height Z-score, WISC – Wechsler Intelligence Scale for children, d – day(s), mo – month(s), y – year(s)

### Risk of bias

Among the 44 articles based on RCTs, 40 were judged to have a high risk of bias, and four were judged to have some concerns (**Table S2** in the **Online Supplementary Document****)**. The primary sources of bias for RCTs included bias in selection of the reported results (32 had a high risk of bias), bias arising from the randomization process (20 had a high risk of bias), and bias due to missing outcome data (18 had a high risk of bias). Among the 13 articles based on CBAs, 11 were judged to have a high risk of bias, one was judged to have a low risk of bias [84], and one was judged to have some concerns [85]. The primary sources of bias for CBAs included bias due to confounding (10 had a high risk of bias), bias in selection of the reported results (seven had a high risk of bias), bias due to missing data (five had a high risk of bias), and bias in selection of participants into the study (five had a high risk of bias).

### Meta-analyses

Nine outcomes were included in three or more independent studies with sufficiently consistent outcome definitions, allowing for meta-analyses. These outcomes were: 1) height; 2) HAZ; 3) weight; 4) WAZ; 5) BAZ; 6) hemoglobin concentrations; 7) plasma/serum ferritin concentrations; 8) mathematical or arithmetic skills; and 9) school attendance. Nineteen studies were included in any of the meta-analyses [29,35,36,52,53,56,57,60,62,65,66,68,69,71-73,76,78,85]. The main reasons for outcomes being excluded from meta-analyses were failure to report quantitative results, lack of variance estimates, reported estimates not able to be synthesized with other studies, or outcomes being included in only one or two studies.

#### Height

**Figure 2:**
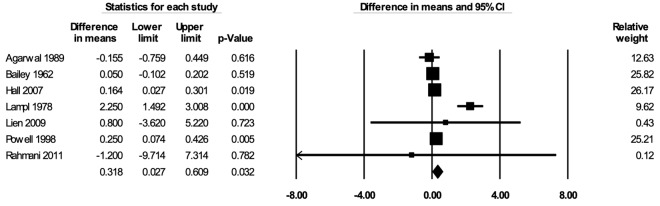
Random-effects meta-analysis of school feeding interventions on 12-month height gain (in centimeters), comparing the group receiving school feeding intervention to the control group. *I^2^* = 82.02%.

The meta-analysis for height included seven studies [29,36,53,57,73,76,78]. In the random-effects meta-analysis including all seven studies (**Figure 2**), school feeding resulted in a statistically significant increase in height; participants who received school feeding gained, on average, 0.318 (95% CI = 0.027, 0.609; *P* = 0.032) centimeters greater heights over 12 months compared to the those in the control groups. No publication bias was detected using the funnel plot (Figure S1 in the **Online Supplementary Document**). The positive effect on height was robust to the removal of any particular study; the estimates with one study removed ranged from 0.141 (95% CI = 0.054, 0.228; *P* = 0.002) centimeters with Lampl 1978 removed to 0.463 (95% CI = 0.043, 0.883; *P* = 0.031) centimeters with Bailey 1962 removed. The effect on height was stronger in RCTs (mean difference = 0.858; 95% CI = 0.312, 1.403; *P* = 0.002) than in CBAs (mean difference = 0.050; 95% CI = -0.359, 0.460; *P* = 0.81) (Figure S2 in the **Online Supplementary Document**), and stronger when formal meals (breakfast, lunch, or dinner) were provided (mean difference = 0.586; 95% CI = 0.109, 1.063; *P* = 0.016) than when snacks were provided (mean difference = 0.111; 95% CI = -0.362, 0.584; *P* = 0.65) (Figure S3 in the **Online Supplementary Document**). The certainty of the evidence for height was rated as moderate.

#### Height-for-age Z-score

**Figure 3:**
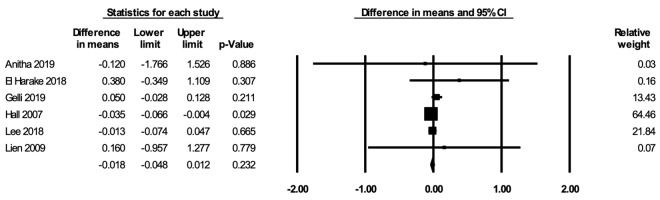
Random-effects meta-analysis of school feeding interventions on 12-month change in height-for-age Z-score, comparing the group receiving school feeding intervention to the control group. *I^2^* = 4.85%.

The meta-analysis for HAZ included six studies [53,69,71,72,78,85]. In the random-effects meta-analysis including all six studies (**Figure 3**), school feeding did not have a statistically significant effect on HAZ (mean difference over 12 months = -0.018; 95% CI = -0.048, 0.012; *P* = 0.23), and the results were robust to the removal of any particular study. No publication bias was detected using the funnel plot (Figure S4 in the **Online Supplementary Document**). The certainty of the evidence for HAZ was rated as moderate.

#### Weight

**Figure 4:**
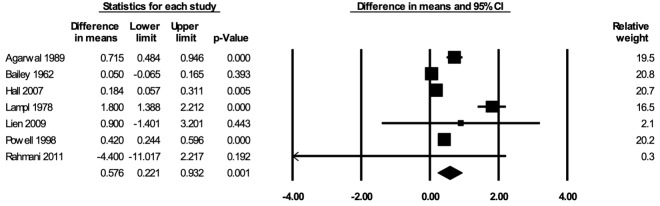
Random-effects meta-analysis of school feeding interventions on 12-month weight gain (in kilograms), comparing the group receiving school feeding intervention to the control group. *I^2^* = 93.20%.

The meta-analysis for weight included seven studies [29,36,53,57,73,76,78]. In the random-effects meta-analysis including all seven studies (**Figure 4**), school feeding resulted in a statistically significant increase in weight; participants who received school feeding gained, on average, 0.576 (95% CI = 0.221, 0.932; *P* = 0.001) kilograms more weights over 12 months compared to the those in the control groups. No publication bias was detected using the funnel plot (Figure S5 in the **Online Supplementary Document**). The positive effect on weight was robust to the removal of any particular study; the estimates with one study removed ranged from 0.327 (95% CI = 0.080, 0.573; *P* = 0.009) kilograms with Lampl 1978 removed to 0.721 (95% CI = 0.265, 1.177; *P* = 0.002) kilograms with Bailey 1962 removed. The effect on weight was stronger in RCTs (mean difference = 0.993; 95% CI = 0.433, 1.554; *P* = 0.001) than in CBAs (mean difference = 0.308; 95% CI = -0.140, 0.756; *P* = 0.18) (Figure S6 in the **Online Supplementary Document**), and stronger when formal meals were provided (mean difference = 0.904; 95% CI = 0.521, 1.287; *P* < 0.001) than when snacks were provided (mean difference = 0.123; 95% CI = -0.303, 0.550; *P* = 0.57) (Figure S7 in the **Online Supplementary Document**). The certainty of the evidence for weight was rated as moderate.

#### Weight-for-age Z-score

**Figure 5:**
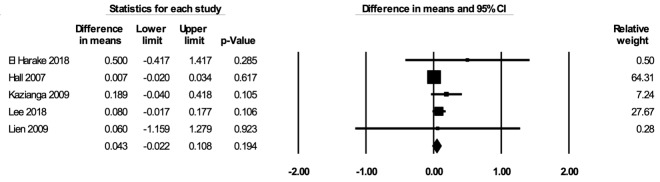
Random-effects meta-analysis of school feeding interventions on 12-month change in weight-for-age Z-score, comparing the group receiving school feeding intervention to the control group. *I^2^* = 25.38%.

The meta-analysis for WAZ included five studies [52,53,69,78,85]. In the random-effects meta-analysis including all five studies (**Figure 5**), school feeding did not have a statistically significant effect on WAZ (mean difference over 12 months = 0.043; 95% CI = -0.022, 0.108; *P* = 0.19). The lack of statistical significance appeared to be due to the non-significant estimates from Hall 2007, which had a large sample size and a greater contribution to the pooled estimate. The effect with Hall 2007 removed was positive and statistically significant (mean difference over 12 months = 0.100; 95% CI = 0.012, 0.189; *P* = 0.027). The asymmetry in the funnel plot suggests a potentially slight selective publication of studies with positive findings (Figure S8 in the **Online Supplementary Document**). The certainty of the evidence for WAZ was rated as low.

#### Body mass index-for-age Z-score

**Figure 6:**
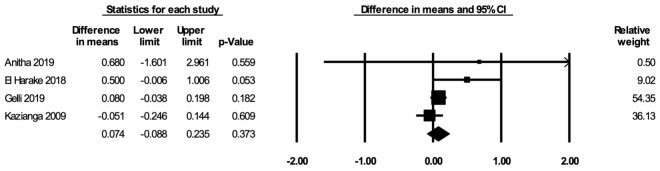
Random-effects meta-analysis of school feeding interventions on 12-month change in body mass index-for-age Z-score, comparing the group receiving school feeding intervention to the control group. *I^2^* = 33.92%.

The meta-analysis for BAZ included four studies [52,71,72,85]. In the random-effects meta-analysis including all four studies (**Figure 6**), school feeding did not have a statistically significant effect on BAZ (mean difference over 12 months = 0.074; 95% CI = -0.088, 0.235; *P* = 0.37), and the results were robust to the removal of any particular study. The asymmetry in the funnel plot suggests a potential selective publication of studies with positive findings (Figure S9 in the **Online Supplementary Document**). The certainty of the evidence for BAZ was rated as low.

#### Hemoglobin concentrations

**Figure 7:**
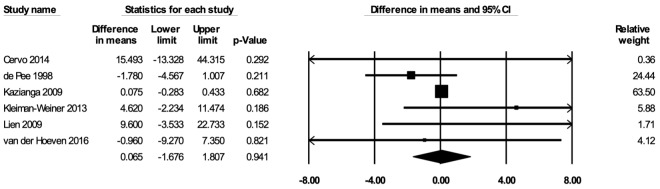
Random-effects meta-analysis of school feeding interventions on 12-month change in hemoglobin concentrations (g/L), comparing the group receiving school feeding intervention to the control group. *I^2^* = 23.87%.

The meta-analysis for hemoglobin concentrations included six studies [35,52,53,62,65,68]. In the random-effects meta-analysis including all six studies (**Figure 7**), school feeding did not have a statistically significant effect on hemoglobin concentrations (mean difference over 12 months = 0.065 g/L; 95% CI = -1.676, 1.807; *P* = 0.94), and the results were robust to the removal of any particular study. No publication bias was detected using the funnel plot (Figure S10 in the **Online Supplementary Document**). The certainty of the evidence for hemoglobin concentrations was rated as low.

#### Plasma/serum ferritin concentrations

**Figure 8:**
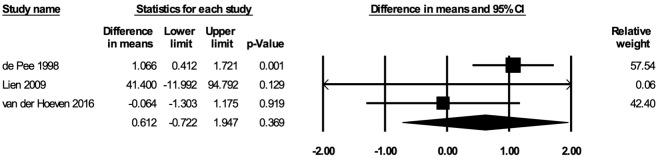
Random-effects meta-analysis of school feeding interventions on 12-month change in plasma or serum ferritin concentrations (μL), comparing the group receiving school feeding intervention to the control group. *I^2^* = 57.62%.

The meta-analysis for plasma or serum ferritin concentrations included three studies [35,53,68]. In the random-effects meta-analysis including all three studies (**Figure 8**), school feeding did not have a statistically significant effect on hemoglobin concentrations (mean difference over 12 months = 0.612 μg/L; 95% CI = -0.722, 1.947; *P* = 0.37), and the results were robust to the removal of any particular study. The certainty of the evidence for plasma or serum ferritin concentrations was rated as low.

#### Mathematical/arithmetic skills

**Figure 9:**
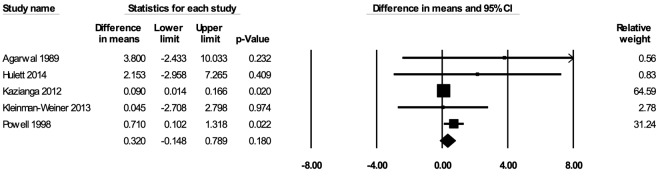
Random-effects meta-analysis of school feeding interventions on 12-month change in mathematical or arithmetic skills, comparing the group receiving school feeding intervention to the control group. *I^2^* = 32.37%.

The meta-analysis for mathematical or arithmetic skills included five studies [36,60,62,66,76]. In the random-effects meta-analysis including all five studies (**Figure 9**), school feeding did not have a statistically significant effect on mathematical/arithmetic skills (mean difference in scores = 0.320; 95% CI = -0.148, 0.789; *P* = 0.18), and the results remained largely robust to the removal of any particular study. No publication bias was detected using the funnel plot (Figure S11 in the **Online Supplementary Document**). The certainty of the evidence for mathematical or arithmetic skills was rated as moderate.

#### School attendance

**Figure 10:**
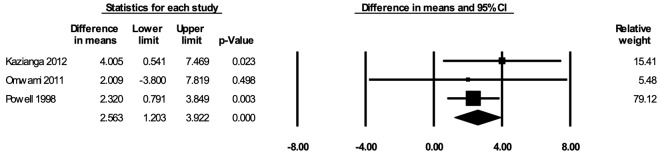
Random-effects meta-analysis of school feeding interventions on 12-month change in school attendance (percentage of possible school days attended), comparing the group receiving school feeding intervention to the control group. *I^2^* = 0.00%.

The meta-analysis for school attendance included three studies [36,56,60]. In the random-effects meta-analysis including all three studies (**Figure 10**), school feeding resulted in a statistically significant increase in the percentage of school days attended; participants who received school feeding attended, on average, 2.56 (95% CI = 1.20, 3.92; *P* < 0.001) percent more school days compared to the those in the control groups. The positive effect on attendance was robust to the removal of any particular study; the estimates with one study removed ranged from 2.30 (95% CI = 0.82, 3.78; *P* = 0.002) percent with Kazianga 2012 removed to 3.48 (95% CI = 0.51, 6.46; *P* = 0.022) percent with Powell 1998 removed. The certainty of the evidence for school attendance was rated as moderate.

## DISCUSSION

We present a systematic review and meta-analysis on the impacts of school feeding interventions on a wide range of health and education outcomes among children and adolescents living in LMICs. We report that school feeding programs confer benefits on height, weight, and school attendance. This work presents an evidence base that will be informative to the design and implementation of school feeding programs targeted toward children and adolescents in resource-constrained settings.

A few systematic or non-systematic reviews have previously synthesized the impacts of school feeding interventions on the health and education of children and adolescents [11-13,86,87]. The only Cochrane systematic review on the impacts of school feeding interventions was published in 2007 and included 18 studies, of which nine were from LMICs [11]. Comprehensive at the time of publication, this Cochrane systematic review included five RCTs conducted in LMICs. More recent reviews on the benefits of school feeding interventions are available but were limited in scope. A qualitative review assessed the impacts of school feeding on the education and health of children in primary schools in LMICs [12]. However, studies conducted at the secondary school level or published before 1990 were not included in this review. Another two reviews [86,87] evaluated interventions for improving education outcomes among children and adolescents in LMICs. Nonetheless, they did not specifically focus on school feeding, nor did they include outcomes related to nutrition and health. The impacts of school feeding on the anthropometry and nutritional status of primary-school-age children and preschool and adolescent girls in LMICs have been reviewed [13], but this work did not specifically examine the impacts on educational outcomes. As more studies become available, updated evidence synthesis on a wide range of outcomes based on systematic literature review is critical.

School meals may provide nutritional benefits for children and adolescents. The benefits of school feeding on anthropometry and school attendance in our review are consistent with those reported in previous work. The estimated benefit of school feeding on weight gain (0.58 kg over 12 months) is similar to the estimates from the Cochrane systematic review [11], which reported that children and adolescents in LMICs receiving school feeding gained an average of 0.25 and 0.75 kg more weight over 12 months in RCTs and CBAs, respectively, compared to those in the control groups. We found a significant effect on height gain (0.32 cm over 12 months) comparing the school feeding group to the control group; this is also generally in line with the slightly mixed results shown in the Cochrane systematic review, which reports non-significant gain in RCTs and a significant gain of 1.43 cm in CBAs [11].

We did not find significant benefits of school feeding on indicators of undernutrition such as HAZ, WAZ, and BAZ. The lack of observed benefits on the anthropometry Z-scores may be due to the small magnitude of the effects (in absolute terms) that might have been insufficient to materially alter the underlying nutritional status or the relative standings in the population. However, most studies were of short or moderate durations, with few studies exceeding 12 months, so they might not have accumulated adequate time for the nutritional benefits of the school feeding interventions to fully manifest. Similarly, the non-significant effects on hemoglobin concentration and ferritin concentrations may be explained by the failure to include iron-rich, animal-source food. When optimally designed considering all the nutritional needs of children and adolescents (beyond merely providing total energy) and provided for an extended period of time, school feeding interventions may have the potential to prevent undernutrition. Future studies should preferably provide formal school meals (as opposed to snacks only) with animal-source food for years and conduct longitudinal follow-up of the participants to better evaluate school feeding programs' long-term benefits.

In addition to the direct nutritional benefits, school feeding has long been used to confer educational benefits. The Cochrane systematic review estimates that children and adolescents receiving school meals attended 4 to 6 more school days per year (in a school year of 172 school days) than those without school meals [11]. Assuming also a school year of 172 days, our estimate of 2.6% more school days attended is equivalent to 4.5 more school days attended, in line with the estimates from the Cochrane systematic review. This finding confirms that school meals can motivate the parents to send their children to school and keep the students in attendance.

The high level of heterogeneity of the school feeding menus and modality indicates that the estimates from the meta-analyses should be interpreted as the average effects; different school feeding programs may well confer quite different levels of benefits. This observation also highlights a historical lack of clear consensus and guidance on the design of school meals in resource-limited settings. It was beyond the scope of the current study to provide definitive recommendations on the design of school feeding programs. However, recent years have seen significant developments in the innovative tools for the development and standardization of school feeding programs in LMICs. For example, the Partnership for Child Development School Meals Planner can be used to create nutritionally balanced and market-costed school meals using daily reference nutrient intake [88]. Similarly, the upcoming PLUS School Menus software will be able to optimize school meals [89]. Based on mathematical optimization algorithms, the PLUS School Menus software calculates the most cost-effective menus, covering the nutritional needs of children from different age groups and optimizing the use of locally sourced food [89]. With global scale-up and capacity building at the country and school levels, tools like these will prove vital for maximizing the potential benefits of school meals in resource-constrained settings. Even with such tools, school feeding interventions nevertheless allow a great deal of flexibility in the design (eg, menu, quantity, timing) based on the specific context; the key elements for successful programs need to be better understood.

A challenge that complicates the interpretation of our results is the generally high risks of bias in previous studies. The primary concerns for both RCT and CBA study designs were the selected reporting of the results and missing data of the outcomes. While school feeding is one of the most long-lasting and well-established nutrition interventions worldwide, it was noted that many studies on school feeding were lacking in the rigor of the methodology or the quality of the reporting. Future studies to evaluate the effectiveness and implementation of school feeding programs should consider the transparent reporting of all pre-specified outcomes, preferably supplemented by a registered protocol. Also, the number of participants with missing data for each outcome (and the reasons for missingness), the potential of selection bias, and measures taken to control for confounding should all be carefully considered and clearly described. Randomized studies should additionally report the process of randomization clearly, so there is no ambiguity on how the intervention received by each study unit (eg, each school) was determined.

Future research priorities may not be whether school feeding impacts nutrition or education in general, but instead on assessing and improving the quality of school feeding programs. Some specific knowledge gaps include identifying appropriate and measurable nutrition indicators, examining school feeding programs by finer age strata, improving the quality and diversity of school meal menus, and strategies for scaling up. The consensus among stakeholders regarding such issues is yet to be reached, and additional studies will contribute valuable insights to the broader research agenda of establishing school feeding guidelines.

The main strengths of this systematic review included the coverage of a wide array of health and education outcomes and the inclusion of all modalities of school feeding. This study has a few potential limitations. First, as most of the included studies had relatively short follow-up periods, we could not make strong inferences regarding the long-term impacts of the school feeding interventions. Second, most of the studies compared school feeding intervention with no intervention at all, which limited our abilities to evaluate whether different contents of school feeding interventions may have different impacts. Third, as a limited number of studies reported process details of the intervention such as the fidelity of the intervention [90] or provided stratified estimates by age or sex, our abilities to conduct subgroup analyses by those characteristics were constrained. Fourth, combining estimates of anthropometry measures across studies conducted among different age groups of children and adolescents was not ideal. However, we also included more age-standardized metrics whenever possible (eg, HAZ, WAZ, and BAZ), which lessened this issue.

In conclusion, this study provides a much-needed synthesis on the impacts of school feeding interventions on the health and education outcomes of children and adolescents living in LMICs. Findings from this study are informative to various stakeholders, including researchers, policymakers, non-governmental organizations, and individual schools, in the design and implementation of school feeding programs. We highlight a need for rigorously designed school feeding studies that have long-term follow-up periods, that are conducted among adolescents in secondary school settings, and that evaluate the impacts on micronutrient deficiencies and academic achievements. Future work is needed to develop clear guidelines and scale up emerging tools for school feeding among children and adolescents in LMICs who are urgently in need of this critical nutrition intervention, especially during the ongoing COVID-19 pandemic.

## Additional material


Online Supplementary Document
