# Supplementary material for: Impacts of school feeding on educational and health outcomes of school-age children and adolescents in low- and middle-income countries: A systematic review and meta-analysis
Source: J Glob Health. 2021 Sep 4;11:04051. doi: 10.7189/jogh.11.04051 (PMC8442580; doi:10.7189/jogh.11.04051)
Supplement: Online Supplementary Document [file jogh-11-04051-s001.pdf]

Table S1 Search strategy of electronic databases

No.	Concept	PubMed search terms	Number of records (As of December 17, 2019)	Embase search terms	Number of records (As of December 17, 2019)	CINAHL search terms	Number of records (As of December 17, 2019)	Cochrane Library search terms	Number of records (As of December 17, 2019)
#1	Randomized controlled trial	randomized controlled trial[pt] OR "randomized controlled trials as topic"[MeSH] OR “controlled trial”[tiab] OR “intervention*”[tiab] OR "random allocation"[MeSH] OR random*[tiab] OR trial*[tiab]	2,213,419	'randomized controlled trial'/exp OR 'controlled trial':ab,ti OR intervention*:ab,ti OR 'randomization'/exp OR random*:ab,ti OR trial*:ab,ti	3,447,902	(MH "Randomized Controlled Trials+") OR TI controlled trial OR AB controlled trial OR TI intervention* OR AB intervention* OR (MH "Random Assignment") OR TI random* OR AB random* OR TI trial* OR AB trial*	745,618	#1 MeSH descriptor: [Randomized Controlled Trial] explode all trees<br>#2 MeSH descriptor: [Randomized Controlled Trials as Topic] explode all trees<br>#3 MeSH descriptor: [Random Allocation] explode all trees<br>#4 ("controlled trial"):ti,ab,kw OR (intervention*):ti,ab,kw OR (random*):ti,ab,kw OR (trial*):ti,ab,kw<br>#5 {OR #1-#4}	1,194,496
#2	Controlled before-after studies	"controlled before-after studies"[MeSH] OR CBA[tiab] OR CBAs[tiab] OR “before-after”[tiab] OR “before after”[tiab] OR “before-and-after”[tiab] OR “before and after”[tiab]	244,320	cba:ab,ti OR cbas:ab,ti OR 'before-after':ab,ti OR 'before after':ab,ti OR 'before-and-after':ab,ti OR 'before and after':ab,ti	367,227	(MH "Controlled Before-After Studies") OR TI CBA OR AB CBA OR TI CBAs OR AB CBAs OR TI before-after OR AB before-after OR TI before after OR AB before after OR TI before-and-after OR AB before-and-after OR TI (before and after) OR AB (before and after)	125,317	#6 MeSH descriptor: [Controlled Before-After Studies] explode all trees<br>#7 (CBA):ti,ab,kw OR (CBAs):ti,ab,kw OR ("before-after"):ti,ab,kw OR ("before after"):ti,ab,kw<br>#8 (before-and-after):ti,ab,kw OR (before and after):ti,ab,kw<br>#9 {OR #6-#8}	162,225
#3	School feeding	("schools"[MeSH] OR school*[tiab] OR "School Health Services"[Mesh]) AND (("meals"[MeSH] OR meal*[tiab]) OR ("lunch"[MeSH] OR lunch*[tiab]) OR ("breakfast"[MeSH] OR breakfast*[tiab]) OR dinner*[tiab] OR ("snacks"[MeSH] OR snack*[tiab]) OR biscuit[tiab] OR ("diet"[MeSH] OR diet[tiab] OR diets[tiab] OR dietary[tiab]) OR ("eating"[MeSH] OR eating[tiab]) OR ("food"[MeSH] OR food*[tiab]) OR (“beverages"[MeSH] OR beverage*[tiab]) OR feed*[tiab] OR "diet, food, and nutrition"[MeSH] OR cater*[tiab] OR cafe*[tiab] OR ("fruit"[MeSH] OR fruit*[tiab]) OR ("vegetables"[MeSH] OR vegetable*[tiab]) OR ("meat"[MeSH] OR meat*[tiab]) OR ("milk"[MeSH] OR milk[tiab]) OR nutritio*[tiab] OR menu*[tiab] OR canteen*[tiab] OR (“gardens”[MeSH] or garden*[tiab]) OR ("food assistance"[MeSH] OR stamp*[tiab] OR voucher*[tiab]))	37,814	('school'/exp OR school*:ab,ti OR 'school health service'/exp) AND ('meal'/exp OR meal*:ab,ti OR lunch*:ab,ti OR breakfast*:ab,ti OR dinner*:ab,ti OR snack*:ab,ti OR 'biscuit'/exp OR biscuit:ab,ti OR 'diet'/exp OR diet:ab,ti OR diets:ab,ti OR dietary:ab,ti OR 'eating'/exp OR eating:ab,ti OR 'food'/exp OR food*:ab,ti OR 'beverage'/exp OR beverage*:ab,ti OR 'feeding'/exp OR feed*:ab,ti OR 'nutrition'/exp OR 'catering service'/exp OR cater*:ab,ti OR 'cafeteria diet'/exp OR cafe*:ab,ti OR 'fruit'/exp OR fruit*:ab,ti OR 'vegetable'/exp OR vegetable*:ab,ti OR 'meat'/exp OR meat*:ab,ti OR 'milk'/exp OR milk:ab,ti OR nutritio*:ab,ti OR menu*:ab,ti OR canteen*:ab,ti OR garden*:ab,ti OR 'food assistance'/exp OR stamp*:ab,ti OR voucher*:ab,ti)	85,338	((MH “schools+”) OR TI school* OR AB school* OR (MH “School Health Services+”) OR (MH “Student Health Services+”) OR (MH "Adolescent Health Services")) AND ((MH “Meals+”) OR TI meal* OR AB meal* OR (MH "Lunch") OR TI lunch* OR AB lunch* OR (MH "Breakfast") OR TI breakfast* OR AB breakfast* OR TI dinner* OR AB dinner* OR (MH "Snacks") OR TI snack* OR AB snack* OR TI biscuit OR AB biscuit OR (MH “Diet+”) OR TI diet OR AB diet OR TI diets OR AB diets OR TI dietary OR AB dietary OR (MH "Eating") OR TI eating OR AB eating OR (MH “Food+”) OR TI food* OR AB food* OR (MH “Beverages+”) OR TI beverage* OR AB beverage* OR TI feed* OR AB feed* OR (MH “Nutrition+”) OR (MH "Public Health Nutrition") OR (MH “Nutrition Services+”) OR TI cater* OR AB cater* OR TI cafe* OR AB cafe* OR (MH “Fruit+”) OR TI fruit* OR AB fruit* OR (MH “Vegetables+”) OR TI vegetable* OR AB vegetable* OR (MH “Meat+”) OR TI meat* OR AB meat* OR (MH “Milk+”) OR TI milk OR AB milk OR TI nutritio* OR AB nutritio* OR (MH "Menu Planning") OR TI menu* OR AB menu* OR TI canteen* OR AB canteen* OR TI garden* OR AB garden* OR (MH "Food Assistance") OR TI stamp* OR AB stamp* OR TI voucher* OR AB voucher*)	19,463	#10 MeSH descriptor: [Schools] explode all trees<br>#11 (school*):ti,ab,kw<br>#12 MeSH descriptor: [School Health Services] explode all trees<br>#13 #10 OR #11 OR #12<br>#14 MeSH descriptor: [Meals] explode all trees<br>#15 (meal*):ti,ab,kw<br>#16 MeSH descriptor: [Lunch] explode all trees<br>#17 (lunch*):ti,ab,kw<br>#18 MeSH descriptor: [Breakfast] explode all trees<br>#19 (breakfast*):ti,ab,kw OR (dinner*):ti,ab,kw<br>#20 MeSH descriptor: [Snacks] explode all trees<br>#21 (snack*):ti,ab,kw OR (biscuit):ti,ab,kw<br>#22 MeSH descriptor: [Diet] explode all trees<br>#23 (diet):ti,ab,kw OR (diets):ti,ab,kw OR (dietary):ti,ab,kw<br>#24 MeSH descriptor: [Eating] explode all trees<br>#25 (eating):ti,ab,kw<br>#26 MeSH descriptor: [Food] explode all trees<br>#27 (food*):ti,ab,kw<br>#28 MeSH descriptor: [Beverages] explode all trees<br>#29 (beverage*):ti,ab,kw OR (feed*):ti,ab,kw<br>#30 MeSH descriptor: [Diet, Food, and Nutrition] explode all trees<br>#31 (cater*):ti,ab,kw OR (cafe*):ti,ab,kw<br>#32 MeSH descriptor: [Fruit] explode all trees<br>#33 (fruit*):ti,ab,kw<br>#34 MeSH descriptor: [Vegetables] explode all trees<br>#35 (vegetable*):ti,ab,kw<br>#36 MeSH descriptor: [Meat] explode all trees<br>#37 (meat*):ti,ab,kw<br>#38 MeSH descriptor: [Milk] explode all trees<br>#39 (milk*):ti,ab,kw<br>#40 (nutritio*):ti,ab,kw OR (menu*):ti,ab,kw OR (canteen*):ti,ab,kw<br>#41 MeSH descriptor: [Gardening] explode all trees<br>#42 (garden*):ti,ab,kw<br>#43 MeSH descriptor: [Food Assistance] explode all trees<br>#44 (stamp*):ti,ab,kw OR (voucher*):ti,ab,kw<br>#45 {OR #14-#44}<br>#46 #13 AND #45	6,783
#4	Children or adolescents	“child”[MeSH] OR “students”[MeSH] OR “adolescent”[MeSH] OR child[tiab] OR children[tiab] OR schoolchildren[tiab] OR kids[tiab] OR kid[tiab] OR student*[tiab] OR teen*[tiab] OR adolescen*[tiab] OR preteen*[tiab]	3,519,775	'child'/exp OR 'student'/exp OR 'adolescent'/exp OR 'adolescence'/exp OR child:ab,ti OR children:ab,ti OR 'school child'/exp OR schoolchildren:ab,ti OR kids:ab,ti OR kid:ab,ti OR student*:ab,ti OR teen*:ab,ti OR adolescen*:ab,ti OR preteen*:ab,ti	4,440,768	(MH “Child+”) OR (MH “Students+”) OR (MH “Adolescence+”) OR TI child OR AB child OR TI children OR AB children OR TI schoolchildren OR AB schoolchildren OR TI kids OR AB kids OR TI kid OR AB kid OR TI student* OR AB student* OR TI teen* OR AB teen* OR TI adolescen* OR AB adolescen* OR TI preteen* OR AB preteen*	1,138,295	#47 MeSH descriptor: [Child] explode all trees<br>#48 MeSH descriptor: [Students] explode all trees<br>#49 MeSH descriptor: [Adolescent] explode all trees<br>#50 (child):ti,ab,kw OR (children):ti,ab,kw OR (schoolchildren):ti,ab,kw OR (kids):ti,ab,kw OR (kid):ti,ab,kw<br>#51 (student*):ti,ab,kw OR (teen*):ti,ab,kw OR (adolescen*):ti,ab,kw OR (preteen*):ti,ab,kw<br>#52 {OR #47-#51}	248,648
#5	Low- and middle-income countries	"Developing Countries"[MeSH] OR developing countr*[tiab] OR developing nation*[tiab] OR less developed countr*[tiab] OR less developed nation*[tiab] OR third world nation*[tiab] OR third world countr*[tiab] OR under developed nation*[tiab] OR underdeveloped nation*[tiab] OR under developed countr*[tiab] OR underdeveloped countr*[tiab] OR middle income countr*[tiab] OR middle income nation*[tiab] OR low income countr*[tiab] OR low income nation*[tiab] OR poor countr*[tiab] OR poor nation*[tiab] OR lmic[tiab] OR lmics[tiab] OR "Africa"[MeSH] OR "Asia"[MeSH] OR "South America"[MeSH] OR "Latin America"[MeSH] OR "Central America"[MeSH] OR africa[tiab] OR asia[tiab] OR south america*[tiab] OR latin america*[tiab] OR central america*[tiab] OR Afghanistan*[tiab] OR Albania*[tiab] OR Algeria*[tiab] OR Samoa*[tiab] OR Angola*[tiab] OR Armenia*[tiab] OR Azerbaijan*[tiab] OR Bangladesh*[tiab] OR Bengali[tiab] OR Belarus*[tiab] OR Belize[tiab] OR Benin[tiab] OR Bhutan*[tiab] OR Bolivia*[tiab] OR Bosnia*[tiab] OR Herzegovina*[tiab] OR Botswana*[tiab] OR Brazil*[tiab] OR Bulgaria*[tiab] OR Burkina Faso[tiab] OR Burundi*[tiab] OR Cabo Verd*[tiab] OR Cape Verd*[tiab] OR Cambodia*[tiab] OR Cameroon*[tiab] OR Central African*[tiab] OR Chad*[tiab] OR China[tiab] OR Chinese[tiab] OR Colombia*[tiab] OR Comoros[tiab] OR Congo[tiab] OR Cook Islands[tiab] OR Costa Rica*[tiab] OR Cote d'Ivoire[tiab] OR Ivory Coast[tiab] OR Cuba[tiab] OR Cuban[tiab] OR Djibouti[tiab] OR Dominica*[tiab] OR Ecuador[tiab] OR Egypt[tiab] OR El Salvador*[tiab] OR Eritrea*[tiab] OR Ethiopia*[tiab] OR Falkland Islands[tiab] OR Fiji*[tiab] OR Gabon*[tiab] OR Gambia*[tiab] OR Georgia*[tiab] OR Ghana*[tiab] OR Grenada*[tiab] OR Guadeloupe[tiab] OR Guatemala*[tiab] OR Guian*[tiab] OR Guinea*[tiab] OR Guyan*[tiab] OR Haiti*[tiab] OR Hondura*[tiab] OR India[tiab] OR Indian*[tiab] OR Indonesia*[tiab] OR Iran*[tiab] OR Iraq*[tiab] OR Jamaica*[tiab] OR Jordan*[tiab] OR Kazakh*[tiab] OR Kenya*[tiab] OR Kiribati[tiab] OR People's Republic of Korea[tiab] OR North Korea[tiab] OR Kosovo[tiab] OR Kosovar*[tiab] OR Kyrgyz*[tiab] OR Lao[tiab] OR Laos[tiab] OR Laotian*[tiab] OR Lebanon[tiab] OR Lebanes*[tiab] OR Lesotho[tiab] OR Liberia*[tiab] OR Libya*[tiab] OR Macedonia*[tiab] OR Madagascar*[tiab] OR Malawi*[tiab] OR Malvinas[tiab] OR Malaysia*[tiab] OR Maldives[tiab] OR Mali[tiab] OR Marshall Island*[tiab] OR Mauritania*[tiab] OR Mauriti*[tiab] OR Mayotte[tiab] OR Mexico[MeSH] OR Mexican*[tiab] OR Micronesia*[tiab] OR Moldova*[tiab] OR Mongolia*[tiab] OR Montenegr*[tiab] OR Montserrat[tiab] OR Morocc*[tiab] OR Mozambique[tiab] OR Myanmar[tiab] OR Burmese*[tiab] OR Burma[tiab] OR Namibia*[tiab] OR Nauru[tiab] OR Nepal*[tiab] OR Netherlands Antilles[tiab] OR Nicaragua*[tiab] OR Niger*[tiab] OR Niue[tiab] OR Pakistan*[tiab] OR Paraguay*[tiab] OR Peru*[tiab] OR Philippin*[tiab] OR Pitcairn[tiab] OR Romania*[tiab] OR Rwanda*[tiab] OR Sao Tome[tiab] OR Principe[tiab] OR Senegal*[tiab] OR Serbia*[tiab] OR Sierra Leone*[tiab] OR Solomon Island*[tiab] OR Somalia*[tiab] OR South Africa*[tiab] OR Sri Lanka[tiab] OR St Helena[tiab] OR Saint Helena[tiab] OR St Lucia[tiab] OR Saint Lucia[tiab] OR St Vincent[tiab] OR Saint Vincent[tiab] OR Grenad*[tiab] OR Sudan*[tiab] OR Suriname*[tiab] OR Swaziland*[tiab] OR Eswatini*[tiab] OR Syria*[tiab] OR Tajik*[tiab] OR Tanzania*[tiab] OR Thai*[tiab] OR Timor*[tiab] OR Togo*[tiab] OR Tokelau[tiab] OR Tonga*[tiab] OR Tunisia*[tiab] OR Turkey[tiab] OR Turkish[tiab] OR Turkmen*[tiab] OR Tuvalu*[tiab] OR Uganda*[tiab] OR Ukrain*[tiab] OR Uzbeki*[tiab] OR Vanuatu*[tiab] OR Venezuela*[tiab] OR Vietnam*[tiab] OR Viet nam*[tiab] OR West Bank[tiab] OR Gaza*[tiab] OR Palestin*[tiab] OR Wallis and Futuna[tiab] OR Yemen*[tiab] OR Zambia*[tiab] OR Zimbabw*[tiab] OR Western Sahara[tiab] OR Argentin*[tiab] OR Russia*[tiab]	2,034,763	'developing country'/exp OR ‘developing countr*’:ab,ti OR ‘developing nation*’:ab,ti OR ‘less developed countr*’:ab,ti OR ‘less developed nation*’:ab,ti OR ‘third world nation*’:ab,ti OR ‘third world countr*’:ab,ti OR ‘under developed nation*’:ab,ti OR ‘underdeveloped nation*’:ab,ti OR ‘under developed countr*’:ab,ti OR ‘underdeveloped countr*’:ab,ti OR 'low income country'/exp OR 'middle income country'/exp OR ‘middle income countr*’:ab,ti OR ‘middle income nation*’:ab,ti OR ‘low income countr*’:ab,ti OR ‘low income nation*’:ab,ti OR ‘poor countr*’:ab,ti OR ‘poor nation*’:ab,ti OR lmic:ab,ti OR lmics:ab,ti OR 'Africa'/exp OR 'African'/exp OR 'Asia'/exp OR 'Asian'/exp OR 'South America'/exp OR 'South American'/exp OR 'Central America'/exp OR 'Central American'/exp OR 'Caribbean Islands'/exp OR africa:ab,ti OR asia:ab,ti OR ‘south america*’:ab,ti OR ‘latin america*’:ab,ti OR ‘central america*’:ab,ti OR Afghanistan*:ab,ti OR 'Albania'/exp OR Albania*:ab,ti OR Algeria*:ab,ti OR 'Samoa'/exp OR 'Samoan Islands'/exp OR 'American Samoa'/exp OR 'Pacific islands'/exp OR 'Atlantic islands'/exp OR Samoa*:ab,ti OR Angola*:ab,ti OR Armenia*:ab,ti OR Azerbaijan*:ab,ti OR Bangladesh*:ab,ti OR Bengali:ab,ti OR Belarus*:ab,ti OR Belize:ab,ti OR Benin:ab,ti OR Bhutan*:ab,ti OR Bolivia*:ab,ti OR 'Bosnia and Herzegovina'/exp OR Bosnia*:ab,ti OR Herzegovina*:ab,ti OR Botswana*:ab,ti OR Brazil*:ab,ti OR 'Bulgaria'/exp OR Bulgaria*:ab,ti OR ‘Burkina Faso’:ab,ti OR Burundi*:ab,ti OR ‘Cabo Verd*’:ab,ti OR ‘Cape Verd*’:ab,ti OR Cambodia*:ab,ti OR Cameroon*:ab,ti OR ‘Central African*’:ab,ti OR Chad*:ab,ti OR China:ab,ti OR Chinese:ab,ti OR Colombia*:ab,ti OR Comoros:ab,ti OR Congo:ab,ti OR ‘Cook Islands’:ab,ti OR ‘Costa Rica*’:ab,ti OR 'cote d`ivoire':ab,ti OR ‘Ivory Coast’:ab,ti OR 'Cuban'/exp OR Cuba:ab,ti OR Cuban:ab,ti OR Djibouti:ab,ti OR Dominica*:ab,ti OR Ecuador:ab,ti OR Egypt:ab,ti OR ‘El Salvador*’:ab,ti OR Eritrea*:ab,ti OR Ethiopia*:ab,ti OR 'Falkland Islands (Malvinas)'/exp OR ‘Falkland Islands’:ab,ti OR Fiji*:ab,ti OR Gabon*:ab,ti OR Gambia*:ab,ti OR 'Georgia (republic)'/exp OR Georgia*:ab,ti OR Ghana*:ab,ti OR Grenada*:ab,ti OR Guadeloupe:ab,ti OR Guatemala*:ab,ti OR Guian*:ab,ti OR Guinea*:ab,ti OR Guyan*:ab,ti OR Haiti*:ab,ti OR Hondura*:ab,ti OR India:ab,ti OR Indian*:ab,ti OR Indonesia*:ab,ti OR Iran*:ab,ti OR Iraq*:ab,ti OR Jamaica*:ab,ti OR Jordan*:ab,ti OR Kazakh*:ab,ti OR Kenya*:ab,ti OR Kiribati:ab,ti OR 'people`s republic of korea':ab,ti OR ‘North Korea’:ab,ti OR 'Kosovo'/exp OR Kosovo:ab,ti OR 'Kosovar'/exp OR Kosovar*:ab,ti OR Kyrgyz*:ab,ti OR Lao:ab,ti OR Laos:ab,ti OR Laotian*:ab,ti OR Lebanon:ab,ti OR Lebanes*:ab,ti OR Lesotho:ab,ti OR Liberia*:ab,ti OR Libya*:ab,ti OR 'Macedonia (republic)'/exp OR Macedonia*:ab,ti OR Madagascar*:ab,ti OR Malawi*:ab,ti OR Malvinas:ab,ti OR Malaysia*:ab,ti OR 'Maldives'/exp OR Maldives:ab,ti OR Mali:ab,ti OR ‘Marshall Island*’:ab,ti OR Mauritania*:ab,ti OR 'Mauritius'/exp OR Mauriti*:ab,ti OR Mayotte:ab,ti OR 'Mexico'/exp OR 'Mexican'/exp OR Mexican*:ab,ti OR Micronesia*:ab,ti OR Moldova*:ab,ti OR Mongolia*:ab,ti OR 'Montenegro (republic)'/exp OR Montenegr*:ab,ti OR Montserrat:ab,ti OR Morocc*:ab,ti OR Mozambique:ab,ti OR Myanmar:ab,ti OR Burmese*:ab,ti OR Burma:ab,ti OR Namibia*:ab,ti OR Nauru:ab,ti OR Nepal*:ab,ti OR ‘Netherlands Antilles’:ab,ti OR Nicaragua*:ab,ti OR Niger*:ab,ti OR Niue:ab,ti OR Pakistan*:ab,ti OR Paraguay*:ab,ti OR Peru*:ab,ti OR Philippin*:ab,ti OR Pitcairn:ab,ti OR 'Romania'/exp OR Romania*:ab,ti OR Rwanda*:ab,ti OR ‘Sao Tome’:ab,ti OR Principe:ab,ti OR Senegal*:ab,ti OR 'Serbia'/exp OR Serbia*:ab,ti OR ‘Sierra Leone*’:ab,ti OR ‘Solomon Island*’:ab,ti OR Somalia*:ab,ti OR ‘South Africa*’:ab,ti OR ‘Sri Lanka’:ab,ti OR ‘St Helena’:ab,ti OR ‘Saint Helena’:ab,ti OR ‘St Lucia’:ab,ti OR ‘Saint Lucia’:ab,ti OR ‘St Vincent’:ab,ti OR ‘Saint Vincent’:ab,ti OR Grenad*:ab,ti OR Sudan*:ab,ti OR Suriname*:ab,ti OR Swaziland*:ab,ti OR Eswatini*:ab,ti OR Syria*:ab,ti OR Tajik*:ab,ti OR Tanzania*:ab,ti OR Thai*:ab,ti OR Timor*:ab,ti OR Togo*:ab,ti OR Tokelau:ab,ti OR Tonga*:ab,ti OR Tunisia*:ab,ti OR Turkey:ab,ti OR Turkish:ab,ti OR Turkmen*:ab,ti OR Tuvalu*:ab,ti OR Uganda*:ab,ti OR Ukrain*:ab,ti OR Uzbeki*:ab,ti OR Vanuatu*:ab,ti OR Venezuela*:ab,ti OR Vietnam*:ab,ti OR ‘Viet nam*’:ab,ti OR ‘West Bank’:ab,ti OR Gaza*:ab,ti OR Palestin*:ab,ti OR ‘Wallis and Futuna’:ab,ti OR Yemen*:ab,ti OR	2,897,904	(MH “Developing Countries+”) OR TI developing countr* OR AB developing countr* OR TI developing nation* OR AB developing nation* OR TI less developed countr* OR AB less developed countr* OR TI less developed nation* OR AB less developed nation* OR TI third world nation* OR AB third world nation* OR TI third world countr* OR AB third world countr* OR TI under developed nation* OR AB under developed nation* OR TI underdeveloped nation* OR AB underdeveloped nation* OR TI under developed countr* OR AB under developed countr* OR TI underdeveloped countr* OR AB underdeveloped countr* OR MH (Low and Middle Income Countries) OR TI middle income countr* OR AB middle income countr* OR TI middle income nation* OR AB middle income nation* OR TI low income countr* OR AB low income countr* OR TI low income nation* OR AB low income nation* OR TI poor countr* OR AB poor countr* OR TI poor nation* OR AB poor nation* OR TI lmic OR AB lmic OR TI lmics OR AB lmics OR (MH “Africa+”) OR (MH "Africa South of the Sahara+") OR (MH “Asia+”) OR (MH “South America+”) OR (MH "Latin America") OR (MH “Central America+”) OR TI africa* OR AB africa* OR TI asia* OR AB asia* OR TI south america* OR AB south america* OR TI latin america* OR AB latin america* OR TI central america* OR AB central america* OR MH Afghanistan OR TI Afghanistan* OR AB Afghanistan* OR MH Albania OR TI Albania* OR AB Albania* OR MH Algeria OR TI Algeria* OR AB Algeria* OR (MH “Samoa+”) OR TI samoa* OR AB samoa* OR MH Angola OR TI Angola* OR AB Angola* OR MH Armenia OR TI Armenia* OR AB Armenia* OR MH Azerbaijan OR TI Azerbaijan* OR AB Azerbaijan* OR MH Bangladesh OR TI Bangladesh* OR AB Bangladesh* OR TI Bengali OR AB Bengali OR MH Byelarus OR TI Belarus* OR AB Belarus* OR MH Belize OR TI Belize OR AB Belize OR MH Benin OR TI Benin OR AB Benin OR MH Bhutan OR TI Bhutan* OR AB Bhutan* OR MH Bolivia OR TI Bolivia* OR AB Bolivia* OR MH Bosnia-Herzegovina OR TI Bosnia* OR AB Bosnia* OR TI Herzegovina* OR AB Herzegovina* OR MH Botswana OR TI Botswana* OR AB Botswana* OR MH Brazil OR TI Brazil* OR AB Brazil* OR MH Bulgaria OR TI Bulgaria* OR AB Bulgaria* OR MH Burkina Faso OR TI Burkina Faso OR AB Burkina Faso OR MH Burundi OR TI Burundi* OR AB Burundi* OR MH Cape Verde OR TI Cabo Verd* OR AB Cabo Verd* OR TI Cape Verd* OR AB Cape Verd* OR MH Cambodia OR TI Cambodia* OR AB Cambodia* OR MH Cameroon OR TI Cameroon* OR AB Cameroon* OR MH Central African Republic OR TI Central African* OR AB Central African* OR MH Chad OR TI Chad* OR AB Chad* OR (MH “China+”) OR TI china OR AB china OR MH Chinese OR TI chinese OR AB chinese OR MH Colombia OR TI Colombia* OR AB Colombia* OR TI Comoros OR AB Comoros OR MH Congo OR MH Democratic Republic of the Congo OR TI Congo OR AB Congo OR TI Cook Islands OR AB Cook Islands OR MH Costa Rica OR TI Costa Rica* OR AB Costa Rica* OR MH Cote d'Ivoire OR TI Cote d'Ivoire OR AB Cote d'Ivoire OR TI Ivory Coast OR AB Ivory Coast OR MH Cuba OR TI Cuba OR AB Cuba OR TI cuban OR AB cuban OR MH Djibouti OR TI Djibouti OR AB Djibouti OR MH Dominica OR TI Dominica* OR AB Dominica* OR MH Ecuador OR TI Ecuador OR AB Ecuador OR MH Egypt OR TI Egypt OR AB Egypt OR MH El Salvador OR TI El Salvador* OR AB El Salvador* OR MH Eritrea OR TI Eritrea* OR AB Eritrea* OR MH Ethiopia OR TI Ethiopia* OR AB Ethiopia* OR TI Falkland Islands OR AB Falkland Islands OR TI Fiji* OR AB Fiji* OR MH Gabon OR TI Gabon* OR AB Gabon* OR MH Gambia OR TI Gambia* OR AB Gambia* OR (MH "Georgia (Republic)") OR TI Georgia* OR AB Georgia* OR MH Ghana OR TI Ghana* OR AB Ghana* OR TI Grenada* OR AB Grenada* OR TI Guadeloupe OR AB Guadeloupe OR MH Guatemala OR TI Guatemala* OR AB Guatemala* OR TI Guian* OR AB Guian* OR MH Guinea OR MH Guinea-Bissau OR MH Papua New Guinea OR MH Equatorial Guinea OR TI Guinea* OR AB Guinea* OR MH Guyana OR TI Guyan* OR AB Guyan* OR MH Haiti OR TI Haiti* OR AB Haiti* OR MH Honduras OR TI Hondura* OR AB Hondura* OR MH India OR TI India OR AB India OR TI Indian* OR AB Indian* OR MH Indonesia OR TI Indonesia* OR AB Indonesia* OR MH Iran OR TI Iran* OR AB Iran* OR MH Iraq OR TI Iraq* OR AB Iraq* OR MH Jamaica OR TI Jamaica* OR AB Jamaica* OR MH Jordan OR TI Jordan* OR AB Jordan* OR MH Kazakhstan OR TI Kazakh* OR AB Kazakh* OR MH Kenya OR TI Kenya* OR AB Kenya* OR TI Kiribati OR AB Kiribati OR MH North Korea OR TI People's Republic of Korea OR AB People's Republic of Korea OR TI North Korea OR AB North Korea OR MH Yugoslavia OR TI Kosovo OR AB Kosovo OR TI Kosovar* OR AB Kosovar* OR TI Kyrgyz* OR AB Kyrgyz* OR MH Laos OR TI Lao OR AB Lao OR TI Laos OR AB Laos OR MH Laotians OR TI Laotian* OR AB Laotian* OR MH Lebanon OR TI Lebanon OR AB Lebanon OR TI Lebanes* OR AB Lebanes* OR MH Lesotho OR TI Lesotho OR AB Lesotho OR MH Liberia OR TI Liberia* OR AB Liberia* OR MH Libya OR TI Libya* OR AB Libya* OR (MH "Macedonia (Republic)") OR TI Macedonia* OR AB Macedonia*	585,553	#53 MeSH descriptor: [Developing Countries] explode all trees<br>#54 ("developing countr*"):ti,ab,kw OR ("developing nation*"):ti,ab,kw OR ("less developed countr*"):ti,ab,kw OR ("less developed nation*"):ti,ab,kw OR ("third world nation*"):ti,ab,kw<br>#55 ("third world countr*"):ti,ab,kw OR ("under developed nation*"):ti,ab,kw OR ("underdeveloped nation*"):ti,ab,kw OR ("under developed countr*"):ti,ab,kw OR ("underdeveloped countr*"):ti,ab,kw<br>#56 ("middle income countr*"):ti,ab,kw OR ("middle income nation*"):ti,ab,kw OR ("low income countr*"):ti,ab,kw OR ("low income nation*"):ti,ab,kw OR ("poor countr*"):ti,ab,kw<br>#57 ("poor nation*"):ti,ab,kw OR (lmic):ti,ab,kw OR (lmics):ti,ab,kw<br>#58 MeSH descriptor: [Africa] explode all trees<br>#59 MeSH descriptor: [Asia] explode all trees<br>#60 MeSH descriptor: [South America] explode all trees<br>#61 MeSH descriptor: [Latin America] explode all trees<br>#62 MeSH descriptor: [Central America] explode all trees<br>#63 (Africa*):ti,ab,kw OR (Asia*):ti,ab,kw OR ("South America*"):ti,ab,kw OR ("Latin America*"):ti,ab,kw OR ("Central America*"):ti,ab,kw<br>#64 (Afghanistan*):ti,ab,kw OR (Albania*):ti,ab,kw OR (Algeria*):ti,ab,kw OR (Samoa*):ti,ab,kw OR (Angola*):ti,ab,kw<br>#65 (Armenia*):ti,ab,kw OR (Azerbaijan*):ti,ab,kw OR (Bangladesh*):ti,ab,kw OR (Bengali):ti,ab,kw OR (Belarus*):ti,ab,kw<br>#66 (Belize):ti,ab,kw OR (Benin):ti,ab,kw OR (Bhutan*):ti,ab,kw OR (Bolivia*):ti,ab,kw OR (Bosnia*):ti,ab,kw<br>#67 (Herzegovina*):ti,ab,kw OR (Botswana*):ti,ab,kw OR (Brazil*):ti,ab,kw OR (Bulgaria*):ti,ab,kw OR ("Burkina Faso"):ti,ab,kw<br>#68 (Burundi*):ti,ab,kw OR ("Cabo Verd*"):ti,ab,kw OR ("Cape Verd*"):ti,ab,kw OR (Cambodia*):ti,ab,kw OR (Cameroon*):ti,ab,kw<br>#69 (Central African*):ti,ab,kw OR (Chad*):ti,ab,kw OR (China):ti,ab,kw OR (Chinese):ti,ab,kw OR (Colombia*):ti,ab,kw<br>#70 (Comoros):ti,ab,kw OR (Congo):ti,ab,kw OR ("Cook Islands"):ti,ab,kw OR ("Costa Rica*"):ti,ab,kw OR ("Cote d'Ivoire"):ti,ab,kw<br>#71 ("Ivory Coast"):ti,ab,kw OR (Cuba):ti,ab,kw OR (Cuban):ti,ab,kw OR (Djibouti):ti,ab,kw OR (Dominica*):ti,ab,kw<br>#72 (Ecuador):ti,ab,kw OR (Egypt):ti,ab,kw OR ("El Salvador*"):ti,ab,kw OR (Eritrea*):ti,ab,kw OR (Ethiopia*):ti,ab,kw<br>#73 ("Falkland Islands"):ti,ab,kw OR (Fiji*):ti,ab,kw OR (Gabon*):ti,ab,kw OR (Gambia*):ti,ab,kw OR (Georgia*):ti,ab,kw<br>#74 (Ghana*):ti,ab,kw OR (Grenada*):ti,ab,kw OR (Guadeloupe):ti,ab,kw OR (Guatemala*):ti,ab,kw OR (Guian*):ti,ab,kw<br>#75 (Guinea*):ti,ab,kw OR (Guyan*):ti,ab,kw OR (Haiti*):ti,ab,kw OR (Hondura*):ti,ab,kw OR (India):ti,ab,kw<br>#76 (Indian*):ti,ab,kw OR (Indonesia*):ti,ab,kw OR (Iran*):ti,ab,kw OR (Iraq*):ti,ab,kw OR (Jamaica*):ti,ab,kw<br>#77 (Jordan*):ti,ab,kw OR (Kazakh*):ti,ab,kw OR (Kenya*):ti,ab,kw OR (Kiribati):ti,ab,kw OR ("People's Republic of Korea"):ti,ab,kw<br>#78 ("North Korea"):ti,ab,kw OR (Kosovo):ti,ab,kw OR (Kosovar*):ti,ab,kw OR (Kyrgyz*):ti,ab,kw OR (Lao):ti,ab,kw<br>#79 (Laos):ti,ab,kw OR (Laotian*):ti,ab,kw OR (Lebanon):ti,ab,kw OR (Lebanes*):ti,ab,kw OR (Lesotho):ti,ab,kw<br>#80 (Liberia*):ti,ab,kw OR (Libya*):ti,ab,kw OR (Macedonia*):ti,ab,kw OR (Madagascar*):ti,ab,kw OR (Malawi*):ti,ab,kw<br>#81 (Malvinas):ti,ab,kw OR (Malaysia*):ti,ab,kw OR (Maldives):ti,ab,kw OR (Mali):ti,ab,kw OR ("Marshall Island*"):ti,ab,kw<br>#82 (Mauritania*):ti,ab,kw OR (Mauriti*):ti,ab,kw OR (Mayotte):ti,ab,kw OR (Mexican*):ti,ab,kw OR (Micronesia*):ti,ab,kw<br>#83 MeSH descriptor: [Mexico] explode all trees	122,277

				Zambia*:ab,ti OR Zimbabw*:ab,ti OR 'Western Sahara':ab,ti OR Argentin*:ab,ti OR 'Russian Federation'/exp OR 'USSR'/exp OR Russia*:ab,ti		OR MH Madagascar OR TI Madagascar* OR AB Madagascar* OR MH Malawi OR TI Malawi* OR AB Malawi* OR TI Malvinas OR AB Malvinas OR MH Malaysia OR TI Malaysia* OR AB Malaysia* OR TI Maldives OR AB Maldives OR MH Mali OR TI Mali OR AB Mali OR TI Marshall Island* OR AB Marshall Island* OR MH Mauritania OR TI Mauritania* OR AB Mauritania* OR TI Mauriti* OR AB Mauriti* OR TI Mayotte OR AB Mayotte OR MH Mexico OR TI Mexico OR AB Mexico OR TI Mexican* OR AB Mexican* OR (MH "Micronesia+") OR TI Micronesia* OR AB Micronesia* OR MH Moldova OR TI Moldova* OR AB Moldova* OR MH Mongolia OR TI Mongolia* OR AB Mongolia* OR TI Montenegr* OR AB Montenegr* OR TI Montserrat OR AB Montserrat OR MH Morocco OR TI Morocc* OR AB Morocc* OR MH Mozambique OR TI Mozambique OR AB Mozambique OR MH Myanmar OR TI Myanmar OR AB Myanmar OR TI Burmese* OR AB Burmese* OR TI Burma OR AB Burma OR MH Namibia OR TI Namibia* OR AB Namibia* OR TI Nauru OR AB Nauru OR MH Nepal OR TI Nepal* OR AB Nepal* OR MH Netherlands Antilles OR TI Netherlands Antilles OR AB Netherlands Antilles OR MH Nicaragua OR TI Nicaragua* OR AB Nicaragua* OR MH Niger OR MH Nigeria OR TI Niger* OR AB Niger* OR TI Niue OR AB Niue OR MH Pakistan OR TI Pakistan* OR AB Pakistan* OR MH Paraguay OR TI Paraguay* OR AB Paraguay* OR Peru OR TI Peru* OR AB Peru* OR MH Philippines OR TI Philippin* OR AB Philippin* OR (MH "Polynesia+") OR TI Pitcairn OR AB Pitcairn OR MH Romania OR TI Romania* OR AB Romania* OR MH Rwanda OR TI Rwanda* OR AB Rwanda* OR TI Sao Tome OR AB Sao Tome OR TI Principe OR AB Principe OR MH Senegal OR TI Senegal* OR AB Senegal* OR MH Serbia OR TI Serbia* OR AB Serbia* OR MH Sierra Leone OR TI Sierra Leone* OR AB Sierra Leone* OR (MH "Pacific Islands+") OR TI Solomon Island* OR AB Solomon Island* OR MH Somalia OR TI Somalia* OR AB Somalia* OR MH South Africa OR TI South Africa* OR AB South Africa* OR MH Sri Lanka OR TI Sri Lanka OR AB Sri Lanka OR TI St Helena OR AB St Helena OR TI Saint Helena OR AB Saint Helena OR TI St Lucia OR AB St Lucia OR TI Saint Lucia OR AB Saint Lucia OR TI St Vincent OR AB St Vincent OR TI Saint Vincent OR AB Saint Vincent OR TI Grenad* OR AB Grenad* OR MH Sudan OR TI Sudan* OR AB Sudan* OR MH Suriname OR TI Suriname* OR AB Suriname* OR MH Swaziland OR TI Swaziland* OR AB Swaziland* OR TI Eswatini* OR AB Eswatini* OR MH Syria OR TI Syria* OR AB Syria* OR MH Tajikistan OR TI Tajik* OR AB Tajik* OR MH Tanzania OR TI Tanzania* OR AB Tanzania* OR MH Thailand OR MH Thai OR TI Thai* OR AB Thai* OR MH Timor OR MH East Timor OR TI Timor* OR AB Timor* OR MH Togo OR TI Togo* OR AB Togo* OR TI Tokelau OR AB Tokelau OR TI Tonga* OR AB Tonga* OR MH Tunisia OR TI Tunisia* OR AB Tunisia* OR MH Turkey OR TI Turkey OR AB Turkey OR TI Turkish OR AB Turkish OR TI Turkmen OR AB Turkmen OR TI Tuvalu* OR AB Tuvalu* OR MH Uganda OR TI Uganda* OR AB Uganda* OR MH Ukraine OR TI Ukrain* OR AB Ukrain* OR MH Uzbekistan OR TI Uzbeki* OR AB Uzbeki* OR TI Vanuatu* OR AB Vanuatu* OR MH Venezuela OR TI Venezuela* OR AB Venezuela* OR MH Vietnam OR TI Vietnam* OR AB Vietnam* OR TI Viet nam* OR AB Viet nam* OR TI West Bank OR AB West Bank OR TI Gaza OR AB Gaza OR TI Palestin* OR AB Palestin* OR TI (Wallis and Futuna) OR AB (Wallis and Futuna) OR MH Yemen OR TI Yemen* OR AB Yemen* OR MH Zambia OR TI Zambia* OR AB Zambia* OR MH Zimbabwe OR TI Zimbabw* OR AB Zimbabw* OR TI Western Sahara OR AB Western Sahara OR MH Argentina OR TI Argentin* OR AB Argentin* OR MH Russia OR TI Russia* OR AB Russia*		#84 (Moldova*):ti,ab,kw OR (Mongolia*):ti,ab,kw OR (Montenegr*):ti,ab,kw OR (Montserrat):ti,ab,kw OR (Morocc*):ti,ab,kw<br>#85 (Mozambique):ti,ab,kw OR (Myanmar):ti,ab,kw OR (Burmese*):ti,ab,kw OR (Burma):ti,ab,kw OR (Namibia*):ti,ab,kw<br>#86 (Nauru):ti,ab,kw OR (Nepal*):ti,ab,kw OR ("Netherlands Antilles"):ti,ab,kw OR (Nicaragua*):ti,ab,kw OR (Niger*):ti,ab,kw<br>#87 (Niue):ti,ab,kw OR (Pakistan*):ti,ab,kw OR (Paraguay*):ti,ab,kw OR (Peru*):ti,ab,kw OR (Philippin*):ti,ab,kw<br>#88 (Pitcairn):ti,ab,kw OR (Romania*):ti,ab,kw OR (Rwanda*):ti,ab,kw OR ("Sao Tome"):ti,ab,kw OR (Principe):ti,ab,kw<br>#89 (Senegal*):ti,ab,kw OR (Serbia*):ti,ab,kw OR ("Sierra Leone*"):ti,ab,kw OR ("Solomon Island*"):ti,ab,kw OR (Somalia*):ti,ab,kw<br>#90 ("South Africa*"):ti,ab,kw OR ("Sri Lanka"):ti,ab,kw OR ("St Helena"):ti,ab,kw OR ("Saint Helena"):ti,ab,kw OR ("St Lucia"):ti,ab,kw<br>#91 ("Saint Lucia"):ti,ab,kw OR ("St Vincent"):ti,ab,kw OR ("Saint Vincent"):ti,ab,kw OR (Grenad*):ti,ab,kw OR (Sudan*):ti,ab,kw<br>#92 (Suriname*):ti,ab,kw OR (Swaziland*):ti,ab,kw OR (Eswatini*):ti,ab,kw OR (Syria*):ti,ab,kw OR (Tajik*):ti,ab,kw<br>#93 (Tanzania*):ti,ab,kw OR (Thai*):ti,ab,kw OR (Timor*):ti,ab,kw OR (Togo*):ti,ab,kw OR (Tokelau):ti,ab,kw<br>#94 (Tonga*):ti,ab,kw OR (Tunisia*):ti,ab,kw OR (Turkey):ti,ab,kw OR (Turkish):ti,ab,kw OR (Turkmen*):ti,ab,kw<br>#95 (Tuvalu*):ti,ab,kw OR (Uganda*):ti,ab,kw OR (Ukrain*):ti,ab,kw OR (Uzbeki*):ti,ab,kw OR (Vanuatu*):ti,ab,kw<br>#96 (Venezuela*):ti,ab,kw OR (Vietnam*):ti,ab,kw OR ("Viet nam*"):ti,ab,kw OR ("West Bank"):ti,ab,kw OR (Gaza*):ti,ab,kw<br>#97 (Palestin*):ti,ab,kw OR ("Wallis and Futuna"):ti,ab,kw OR (Yemen*):ti,ab,kw OR (Zambia*):ti,ab,kw OR (Zimbabw*):ti,ab,kw<br>#98 ("Western Sahara"):ti,ab,kw OR (Argentin*):ti,ab,kw OR (Russia*):ti,ab,kw<br>#99 {OR #53-#98}	
RCTs	#1 AND #3 AND #4 AND #5		2,859		5,792		1,721		1,564
CBAs	#2 AND #3 AND #4 AND #5		166		320		168		226
Total	(#1 OR #2) AND #3 AND #4 AND #5		2,921		5,921		1,791		1,573

Table S2 Risk of bias of the included studies

Study	Randomized trials							Non-randomized studies							
	Bias arising from the randomization process	Bias due to deviation from intended interventions	Bias due to missing outcome data	Bias in measurement of the outcome	Bias in selection of the reported result	For cluster-randomized trials only: Bias arising from the timing of identification and recruitment of individual participants in relation to timing of randomization	Overall bias	Bias due to confounding	Bias due to deviations from intended interventions	Bias due to missing data	Bias in classification of interventions	Bias in measurement of outcomes	Bias in selection of participants into the study	Bias in selection of the reported result	Overall bias
Bailey 1962								Critical	No information	No information	Low	Moderate	No information	No information	Critical
Lampl 1978	High	Some concerns	Low	Some concerns	High		High								
Powell 1983								Serious	Low	No information	Low	Moderate	Moderate	No information	Serious
Spurr 1987								Serious	Low	Low	Low	No information	Low	Serious	Serious
Agarwal 1989								No information	Low	Serious	Serious	No information	Serious	Serious	Serious
Chandler 1995	Some concerns	Low	Some concerns	High	High	Some concerns	High								
Pollitt 1996	Some concerns	Some concerns	Low	Some concerns	High	Low	High								
Jacoby 1996	High	Low	High	Some concerns	High	Low	High								
Chang 1996	Some concerns	Some concerns	High	High	High	High	High								
Richter 1997								Serious	No information	Serious	Serious	Serious	Serious	Serious	Serious
Powell 1998	Some concerns	Low	High	High	Some concerns	High									
Grantham-McGregor 1998	High	Some concerns	High	High	Some concerns	Low	High								
de Pee 1998	Some concerns	Low	High	Low	Some concerns	High									
Tan 1999	High	Low	High	High	High	High	High								
Ma 1999	Some concerns	Some concerns	Low	High	High		High								
Whaley 2003	Some concerns	Some concerns	Low	Some concerns	High	Low	High								
Grillenberger 2003	Some concerns	Low	Low	Some concerns	Some concerns	Some concerns	Some concerns								
Siekmann 2003a	Some concerns	Some concerns	Low	Some concerns	High	Low	High								
Siekmann 2003b	High	High	Low	Some concerns	High	Some concerns	High								
Du 2004	Some concerns	Low	Some concerns	Low	Some concerns	Low	Some concerns								
Sigman 2005	High	Some concerns	Low	Some concerns	High	Low	High								
Zhu 2005	Some concerns	Some concerns	Low	Some concerns	High	Low	High								
Zhu 2006	Some concerns	Some concerns	High	Some concerns	High	Low	High								
Neumann 2007	Some concerns	Low	High	High	High	Low	High								
Hall 2007								Serious	Low	Serious	Low	Moderate	Serious	Serious	Serious
Muthayya 2007	High	Some concerns	Some concerns	Some concerns	High		High								
Zhu 2008	Some concerns	Some concerns	Low	Some concerns	High	Low	High								
Adelman 2008	High	High	High	High	High	Low	High								
Alderman 2008	Some concerns	High	High	High	High	High	High								
Tupe 2009	Some concerns	Low	Low	Low	Some concerns	Some concerns									
Lien 2009	High	Low	Low	Some concerns	High		High								
Alaofe 2009								Serious	Low	Low	Low	Low	Serious	Moderate	Serious
Kazianga 2009	High	Low	Low	High	High	Some concerns	High								
Ohiokpehai 2009								Serious	No information	Serious	Low	No information	No information	Serious	Serious
Mayurasakorn 2010	Some concerns	High	Some concerns	Some concerns	High		High								
Vaz 2011	Some concerns	Some concerns	Low	Low	High		High								
Omwami 2011	High	High	High	High	High	Low	High								
Rahmani 2011	High	Some concerns	Some concerns	High	High	Some concerns	High								
Li 2011	High	Some concerns	High	Some concerns	Some concerns	High									
Alderman 2012	Some concerns	High	High	High	High	High	High								
Kazianga 2012	High	Low	Low	High	High	Some concerns	High								
Kleiman-Weiner 2013	High	Some concerns	Some concerns	Low	Some concerns	Low	High								
Joulaei 2013								Serious	No information	No information	Low	Moderate	Serious	Serious	Serious
Neumann 2013a	High	Low	High	Some concerns	High	High	High								
Neumann 2013b	High	Low	High	Some concerns	High	High	High								
Diagne 2014	High	Some concerns	High	High	High	Some concerns	High								
Hulett 2014	Some concerns	Low	High	Some concerns	High	Low	High								
Cervo 2014	Some concerns	Low	Low	Low	High		High								
Lin 2015								Serious	Low	Moderate	Low	Moderate	Low	Serious	Serious
van der Hoeven 2016	Some concerns	Low	Low	Low	Low		Some concerns								
Adams 2017								Moderate	Low	Moderate	Low	Low	Moderate	No information	Moderate
Baum 2017								Critical	Serious	Serious	Low	moderate	Low	No information	Critical
El Harake 2018								Moderate	Moderate	Low	Low	No information	Low	No information	No information
Lee 2018	Low	Low	Some concerns	Low	High		High								
Gelli 2019	Some concerns	Some concerns	Low	High	Low	Low	High								
Anitha 2019	High	High	High	Low	Some concerns	High	High								
Adelman 2019	High	High	Some concerns	Low	Some concerns	Some concerns	High								

Online Supporting Material

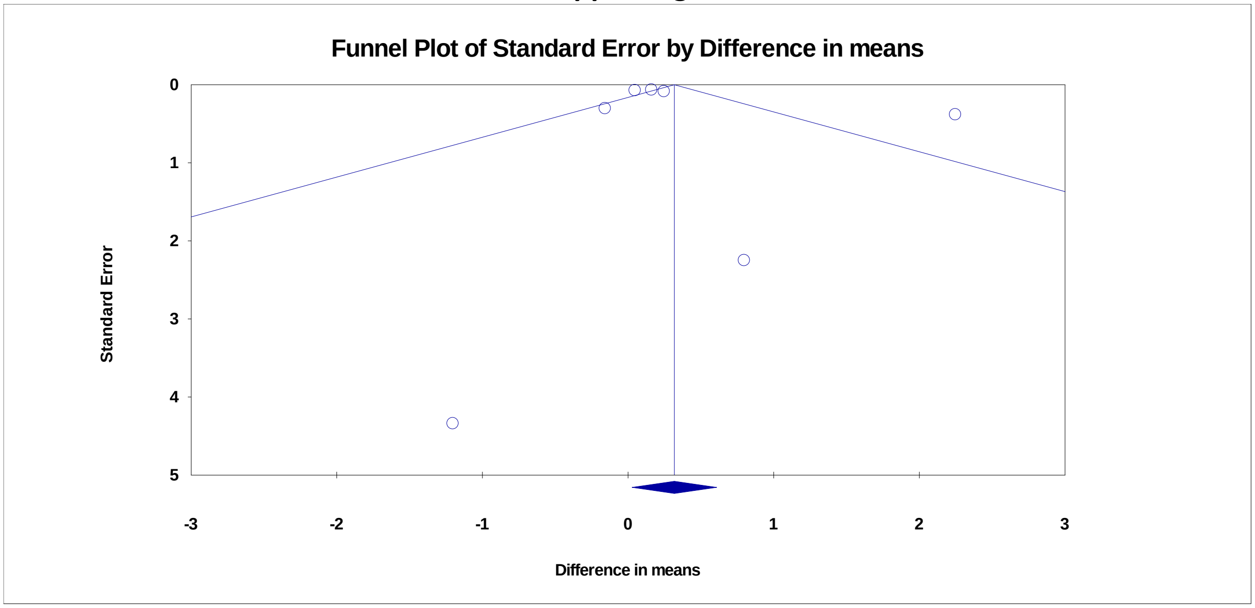

Figure S1 Funnel plot for the random-effects meta-analysis of school feeding interventions on 12-month height gain (in centimeters).

Group by Design	Study name	Statistics for each study: Difference in means	Lower limit	Upper limit	p-Value	Relative weight
CBA	Agarwal 1989	-0.155	-0.759	0.449	0.616	21.71
CBA	Bailey 1962	0.050	-0.102	0.202	0.519	38.95
CBA	Hall 2007	0.164	0.027	0.301	0.019	39.34
CBA		0.050	-0.359	0.460	0.809	
RCT	Lampl 1978	2.250	1.492	3.008	0.000	30.27
RCT	Lien 2009	0.800	-3.620	5.220	0.723	1.49
RCT	Powell 1998	0.250	0.074	0.426	0.005	67.83
RCT	Rahmani 2011	-1.200	-9.714	7.314	0.782	0.41
RCT		0.858	0.312	1.403	0.002	

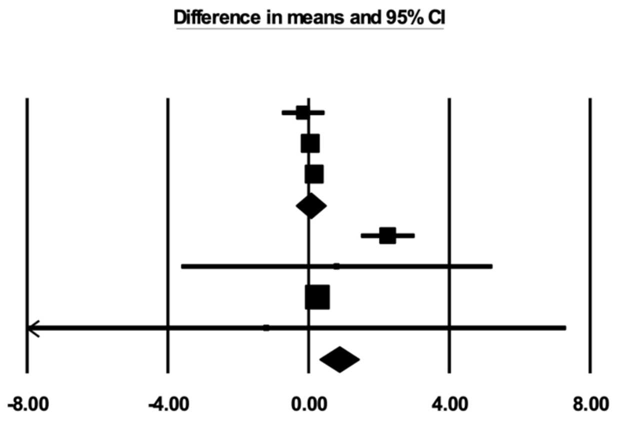

Figure S2 Random-effects meta-analysis of school feeding interventions on 12-month height gain (in centimeters), stratified by study design (CBA or RCT). I^2 = 0.00% among CBAs and 88.24% among RCTs. CBA, controlled before-after study; RCT, randomized controlled trial.

Online Supporting Material

Group by Modality	Study name	Difference in means	Lower limit	Upper limit	p-Value	Relative weight
		Statistics for each study				
Meals	Agarwal 1989	-0.155	-0.759	0.449	0.616	28.49
Meals	Lampl 1978	2.250	1.492	3.008	0.000	22.56
Meals	Powell 1998	0.250	0.074	0.426	0.005	48.96
Meals		0.586	0.109	1.063	0.016	
Snacks	Bailey 1962	0.050	-0.102	0.202	0.519	49.05
Snacks	Hall 2007	0.164	0.027	0.301	0.019	49.52
Snacks	Lien 2009	0.800	-3.620	5.220	0.723	1.12
Snacks	Rahmani 2011	-1.200	-9.714	7.314	0.782	0.31
Snacks		0.111	-0.362	0.584	0.646	

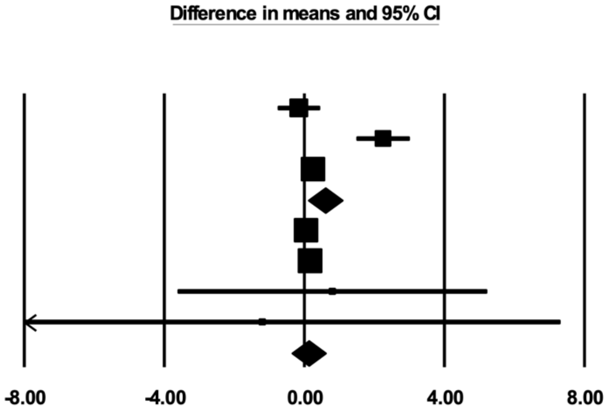

Figure S3 Random-effects meta-analysis of school feeding interventions on 12-month height gain (in centimeters), stratified by feeding modality (formal meals or snacks). I^2 = 92.82% among formal meals and 0.00% among snacks.

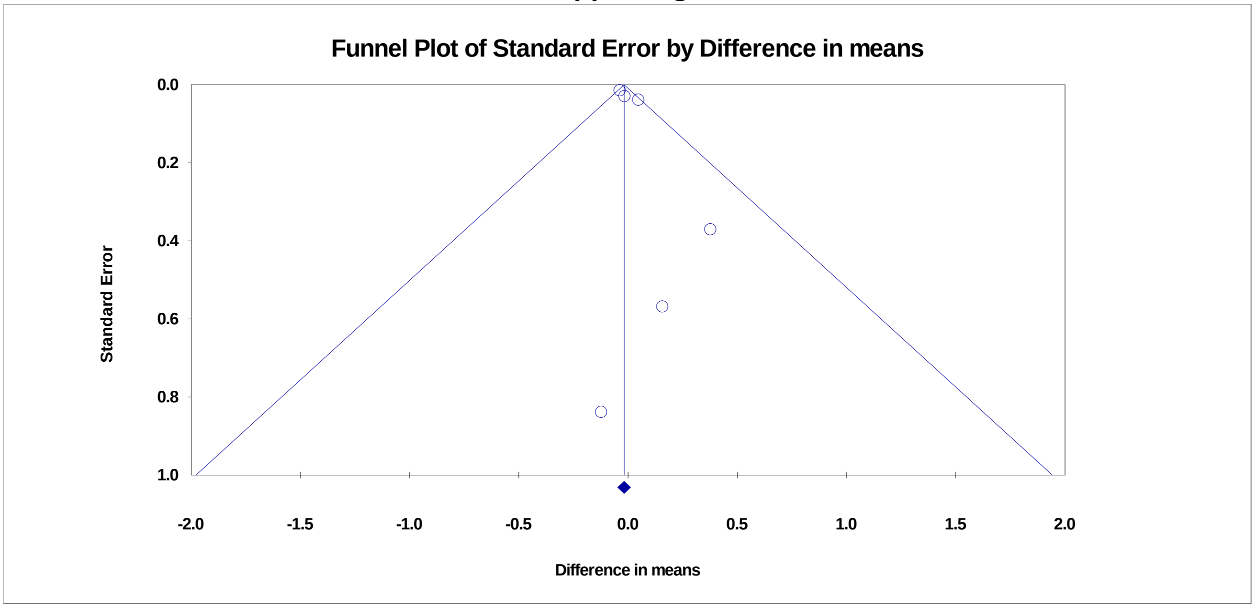

Figure S4 Funnel plot for the random-effects meta-analysis of school feeding interventions on 12-month change in height-for-age Z-score.

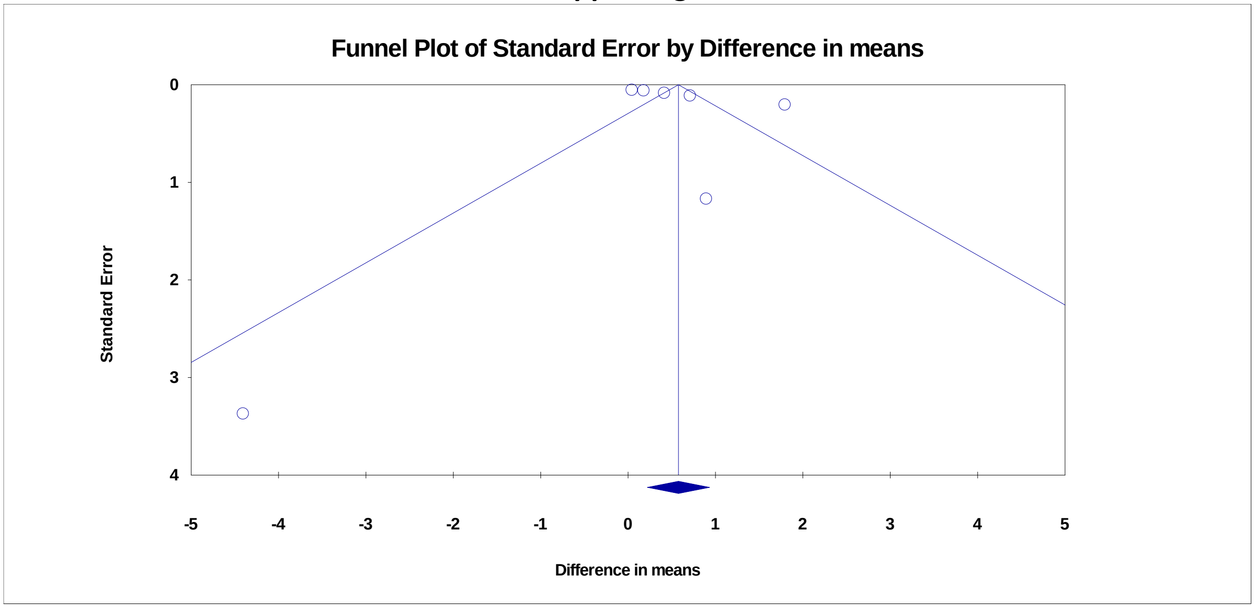

Figure S5 Funnel plot for the random-effects meta-analysis of school feeding interventions on 12-month weight gain (in kilograms).

Online Supporting Material

Group by Design	Study name	Difference in means	Lower limit	Upper limit	p-Value	Relative weight
CBA	Agarwal 1989	0.715	0.484	0.946	0.000	31.9
CBA	Bailey 1962	0.050	-0.065	0.165	0.393	34.1
CBA	Hall 2007	0.184	0.057	0.311	0.005	33.9
CBA		0.308	-0.140	0.756	0.178	
RCT	Lampl 1978	1.800	1.388	2.212	0.000	42.2
RCT	Lien 2009	0.900	-1.401	3.201	0.443	5.3
RCT	Powell 1998	0.420	0.244	0.596	0.000	51.8
RCT	Rahmani 2011	-4.400	-11.017	2.217	0.192	0.7
RCT		0.993	0.433	1.554	0.001	

Statistics for each study

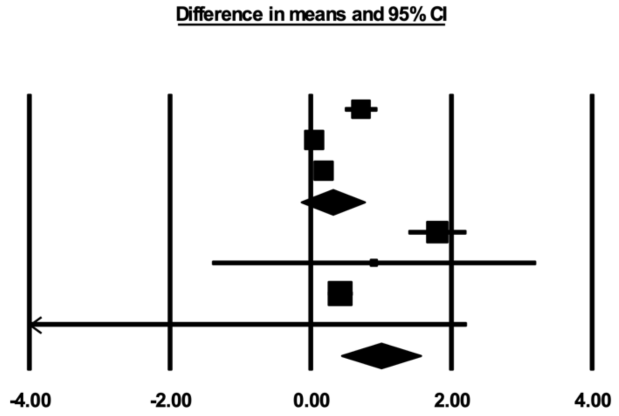

Figure S6 Random-effects meta-analysis of school feeding interventions on 12-month weight gain (in kilograms), stratified by study design (CBA or RCT). I^2 = 92.18% among CBAs and 92.26% among RCTs. CBA, controlled before-after study; RCT, randomized controlled trial.

Group by Modality	Study name	Difference in means	Lower limit	Upper limit	p-Value	Relative weight
Meals	Agarwal 1989	0.715	0.484	0.946	0.000	35.2
Meals	Lampl 1978	1.800	1.388	2.212	0.000	27.6
Meals	Powell 1998	0.420	0.244	0.596	0.000	37.2
Meals		0.904	0.521	1.287	0.000	
Snacks	Bailey 1962	0.050	-0.065	0.165	0.393	48.4
Snacks	Hall 2007	0.184	0.057	0.311	0.005	48.0
Snacks	Lien 2009	0.900	-1.401	3.201	0.443	3.2
Snacks	Rahmani 2011	-4.400	-11.017	2.217	0.192	0.4
Snacks		0.123	-0.303	0.550	0.571	

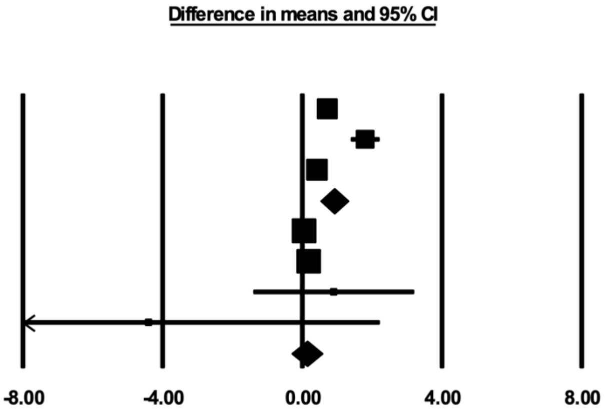

Figure S7 Random-effects meta-analysis of school feeding interventions on 12-month weight gain (in kilograms), stratified by feeding modality (formal meals or snacks). I^2 = 94.57% among formal meals and 34.56% among snacks.

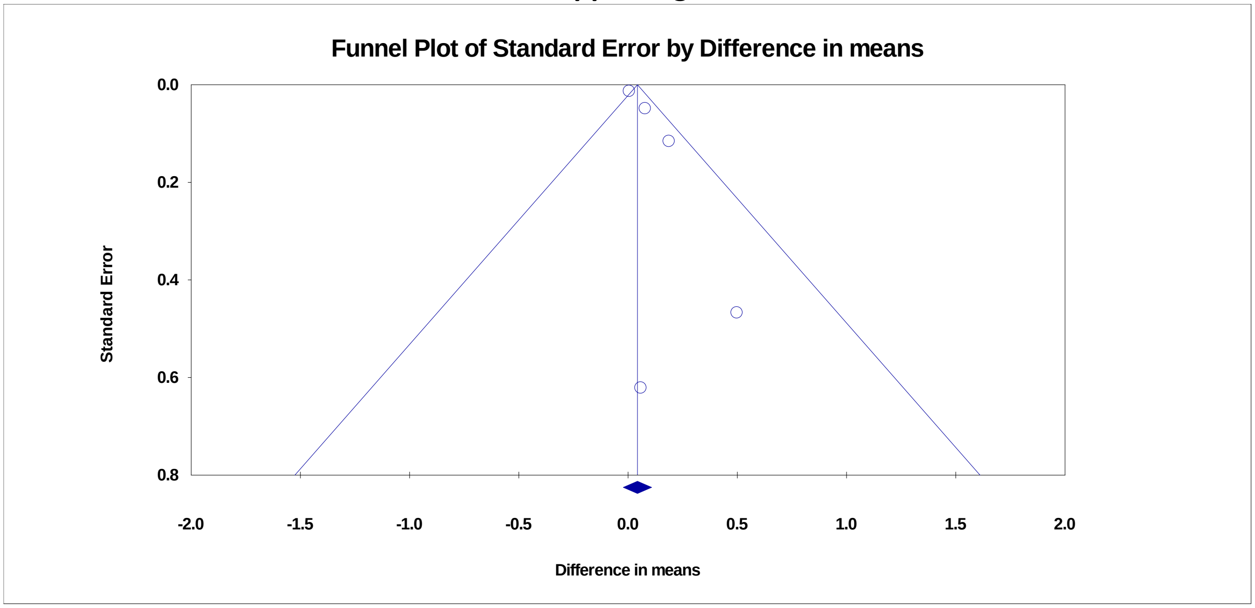

Figure S8 Funnel plot for the random-effects meta-analysis of school feeding interventions on 12-month change in weight-for-age Z-score.

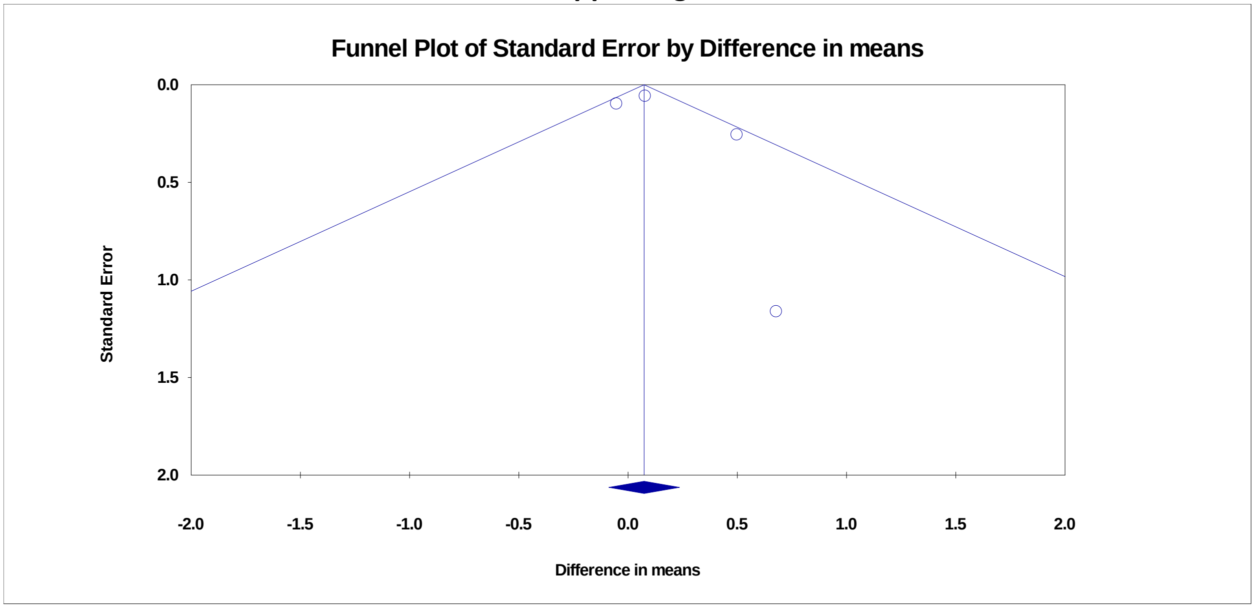

Figure S9 Funnel plot for the random-effects meta-analysis of school feeding interventions on 12-month change in body mass index-for-age Z-score.

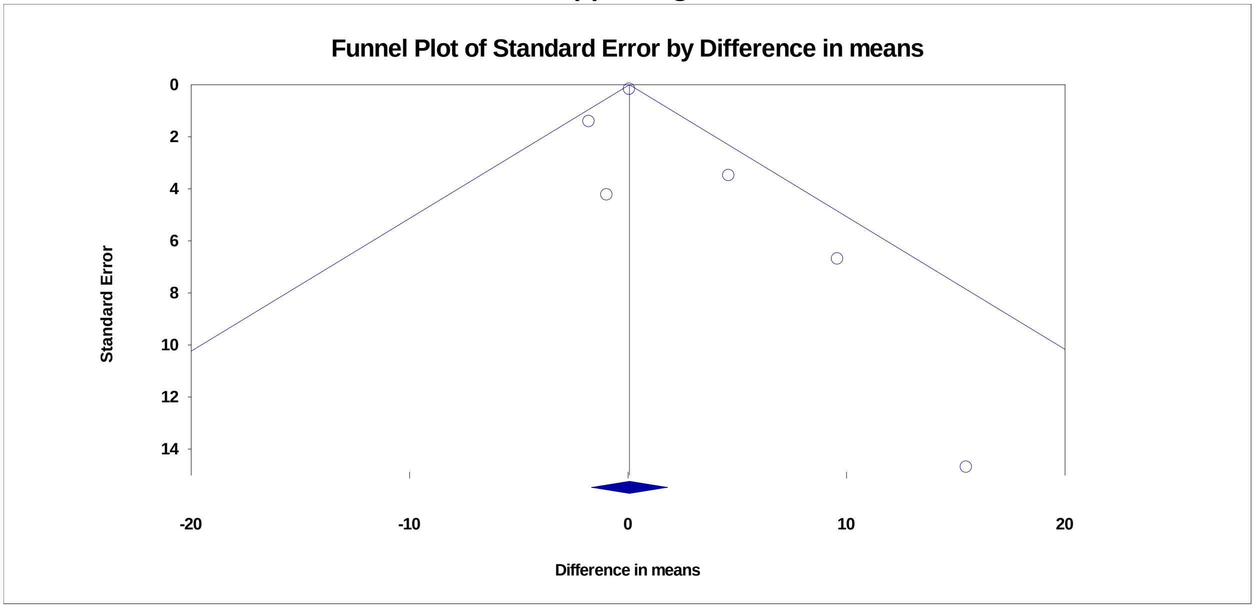

Figure S10 Funnel plot for the random-effects meta-analysis of school feeding interventions on 12-month change in hemoglobin concentrations (g/L).

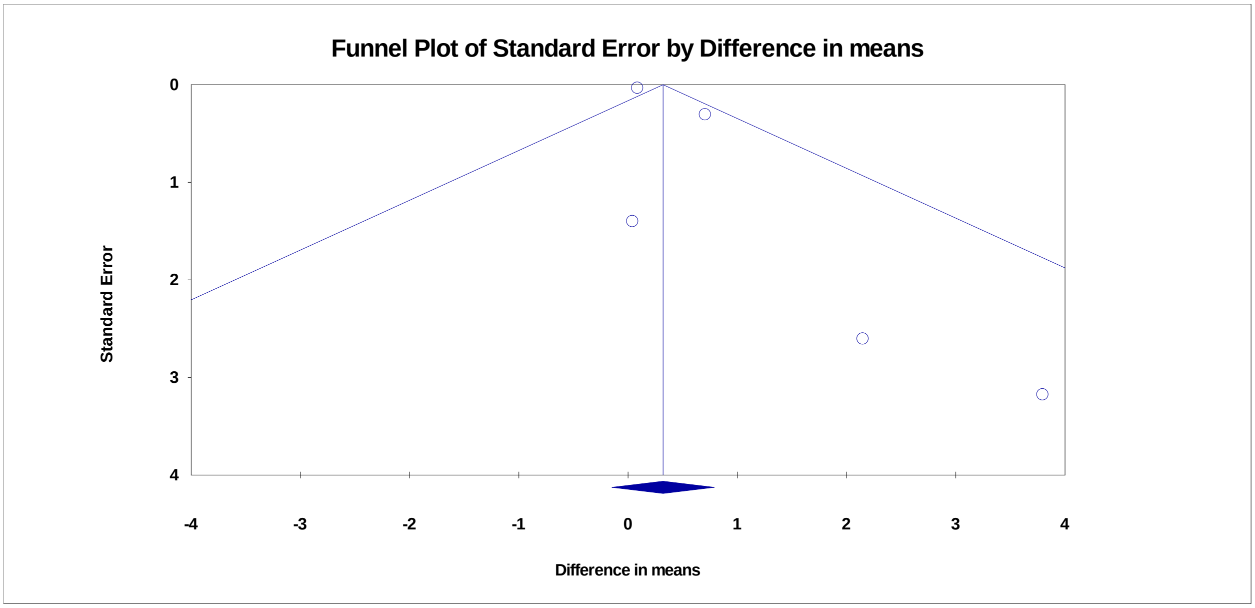

Figure S11 Funnel plot for the random-effects meta-analysis of school feeding interventions on 12-month change in mathematical or arithmetic skills.
